# Meningeal dendritic cells drive neuropathic pain through elevation of the kynurenine metabolic pathway in mice

**DOI:** 10.1172/JCI153805

**Published:** 2022-12-01

**Authors:** Alexandre G. Maganin, Guilherme R. Souza, Miriam D. Fonseca, Alexandre H. Lopes, Rafaela M. Guimarães, André Dagostin, Nerry T. Cecilio, Atlante S. Mendes, William A. Gonçalves, Conceição E.A. Silva, Francisco Isaac Fernandes Gomes, Lucas M. Mauriz Marques, Rangel L. Silva, Letícia M. Arruda, Denis A. Santana, Henrique Lemos, Lei Huang, Marcela Davoli-Ferreira, Danielle Santana-Coelho, Morena B. Sant’Anna, Ricardo Kusuda, Jhimmy Talbot, Gabriela Pacholczyk, Gabriela A. Buqui, Norberto P. Lopes, Jose C. Alves-Filho, Ricardo M. Leão, Jason C. O’Connor, Fernando Q. Cunha, Andrew Mellor, Thiago M. Cunha

**Affiliations:** 1 Center for Research in Inflammatory Diseases (CRID), Department of Pharmacology and; 2 Graduate Program in Basic and Applied Immunology, Ribeirao Preto Medical School, University of Sao Paulo, Ribeirao Preto, Brazil.; 3 Cancer Immunology, Inflammation and Tolerance Program, Cancer Center, Georgia Regents University, Augusta, Georgia, USA.; 4 Department of Pharmacology, University of Texas Health Science Center at San Antonio and Audie L. Murphy VA Hospital, San Antonio, Texas, USA.; 5 NPPNS, Department of Physic and Chemistry, School of Pharmaceutical Sciences of Ribeirão Preto, University of Sao Paulo, Ribeirao Preto, Brazil.; 6 Department of Physiology, Ribeirao Preto Medical School, University of Sao Paulo, Ribeirao Preto, Brazil.

**Keywords:** Metabolism, Neuroscience, Amino acid metabolism, Dendritic cells, Pain

## Abstract

Neuropathic pain is one of the most important clinical consequences of injury to the somatosensory system. Nevertheless, the critical pathophysiological mechanisms involved in neuropathic pain development are poorly understood. In this study, we found that neuropathic pain is abrogated when the kynurenine metabolic pathway (KYNPATH) initiated by the enzyme indoleamine 2,3-dioxygenase 1 (IDO1) is ablated pharmacologically or genetically. Mechanistically, it was found that IDO1-expressing dendritic cells (DCs) accumulated in the dorsal root leptomeninges and led to an increase in kynurenine levels in the spinal cord. In the spinal cord, kynurenine was metabolized by kynurenine-3-monooxygenase–expressing astrocytes into the pronociceptive metabolite 3-hydroxykynurenine. Ultimately, 3-hydroxyanthranilate 3,4-dioxygenase–derived quinolinic acid formed in the final step of the canonical KYNPATH was also involved in neuropathic pain development through the activation of the glutamatergic *N*-methyl-D-aspartate receptor. In conclusion, these data revealed a role for DCs driving neuropathic pain development through elevation of the KYNPATH. This paradigm offers potential new targets for drug development against this type of chronic pain.

## Graphical abstract

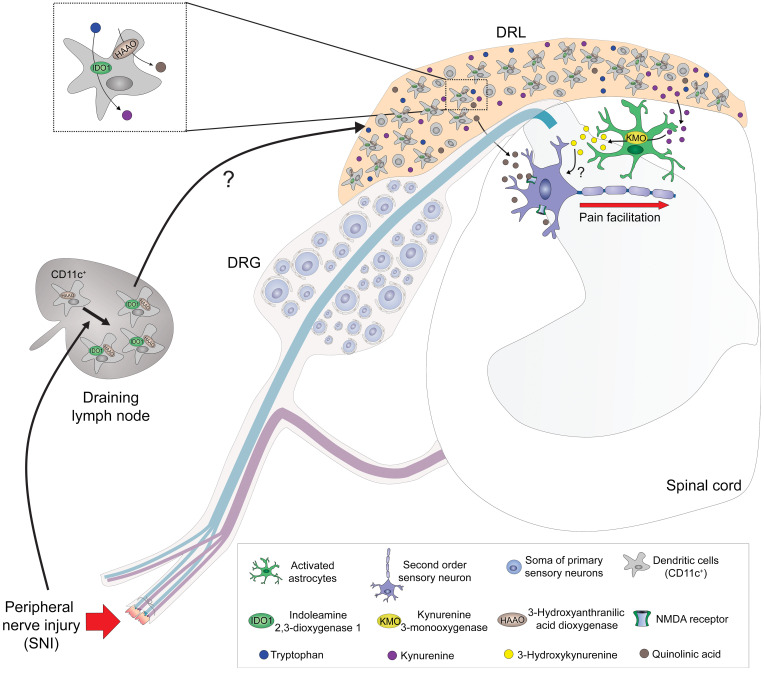

## Introduction

Neuropathic pain is one of the most clinically relevant types of chronic pain. It is generally a consequence of direct damage to the somatosensory nervous system. Although several pathophysiological mechanisms that generate neuropathic pain have been described, developing effective treatments remains a challenge (1). Among the mechanisms involved in the development and maintenance of neuropathic pain, neuron–glial cell (microglia/astrocytes/oligodendrocytes) interactions in the spinal cord seem to play a crucial role in neuropathic pain development through the amplification of central sensitization (2). More specifically, glial cell–derived mediators that enhance glutamatergic transmission in spinal cord neurons are thought to perpetuate neuropathic pain (2, 3).

**Figure 1:**
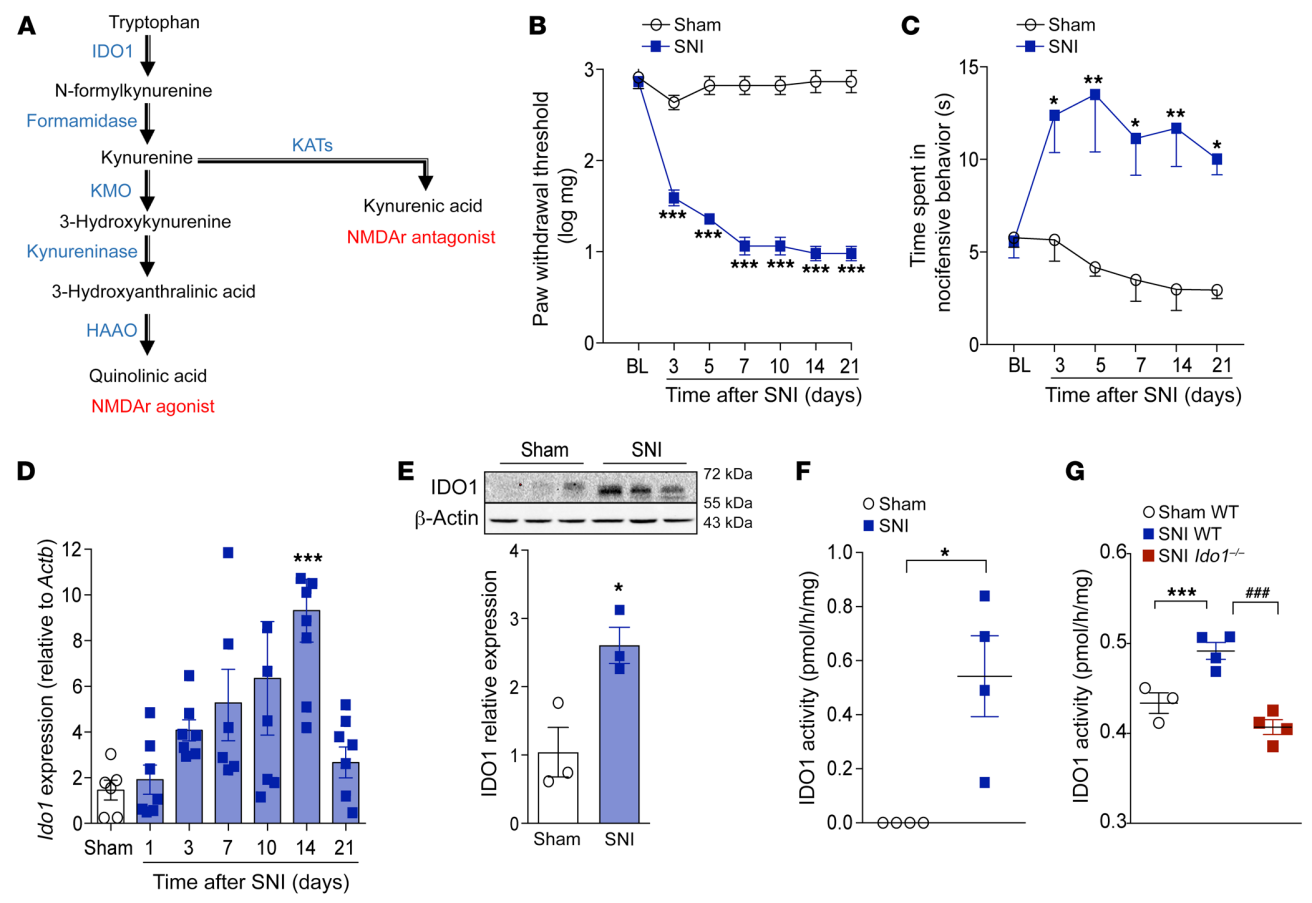
IDO1 expression and activity in spinal cord after peripheral nerve injury. (**A**) Simplified diagram of the kynurenine metabolic pathway. (**B** and **C**) Time course of (**B**) mechanical or (**C**) cold allodynia after spared nerve injury (SNI) model (*n* = 5). (**D**) *Ido1* mRNA expression in the ipsilateral dorsal horn of the spinal cord after sham (14 days) or SNI surgery (3–21 days after injury) (*n* = 6–7). (**E**) Western blotting analysis of IDO1 expression in the ipsilateral dorsal horn of the spinal cord 14 days after sham or SNI surgery (*n* = 3). (**F**) IDO1 enzymatic activity in the ipsilateral dorsal horn of the spinal cords 14 days after sham or SNI surgery in WT (*n* = 4, pooled from 5 mice) and (**G**) compared to *Ido1^–/–^* mice (*n* = 3–4 pooled from 5 mice). Data are expressed as mean ± SEM. *P < 0.05, **P < 0.01, ***P < 0.001 versus sham; ^###^*P* < 0.001 versus *Ido1^–/–^* mice by 2-way ANOVA with Bonferroni’s post hoc test (**B** and **C**), 1-way ANOVA with Bonferroni’s post hoc test (**D** and **G**), or unpaired, 2-tailed Student’s *t* test (**E** and **F**).

The kynurenine (Kyn) metabolic pathway (KYNPATH) is a catabolic system linked to pathophysiological processes (4, 5). For instance, disturbances in KYNPATH have been implicated in several human diseases, such as depression, schizophrenia, and Alzheimer’s and Huntington’s diseases (6, 7). Bioactive kynurenines (including Kyn itself) are generated by indoleamine 2,3-dioxygenase 1–mediated (IDO1-mediated) or tryptophan 2,3-dioxygenase–mediated oxidative catabolism of the essential amino acid tryptophan in peripheral tissues and the central nervous system (CNS), as described in several studies (8–10). In addition, Kyn can be converted to several downstream kynurenine metabolites, such as 3-hydroxykynurenine (3-Hk), 3-hydroxyanthranilic acid (3-Haa), and quinolinic acid (QA), the formation of which is mainly mediated by 2 downstream enzymes, kynurenine-3-monooxygenase (KMO) and 3-hydroxyanthranilic acid dioxygenase (HAAO), respectively, as shown in Figure 1A (11–13). Kynureninase is also involved in the conversion of 3-Hk into 3-Haa, as shown in Figure 1A (5). On the other hand, Kyn can be also metabolized in a side-arm reaction by kynurenine aminotransferases (KATs) into kynurenic acid (Kyna), as shown in Figure 1A (14). These “kynurenines” are biologically active in both the periphery and CNS. The upregulation of the KYNPATH is generally a consequence of immune and glial cell activation in either the periphery or the CNS (4, 15). Elevated levels of kynurenine metabolites in the CNS, especially 3-Hk and QA, might cause neuronal dysfunction and damage, processes that are dependent on an increase in oxidative stress and glutamatergic *N*-methyl-D-aspartate (NMDA) receptor activation, respectively (16, 17). Although participation of KYNPATH in the pathophysiology of neuropathic pain has been investigated in a few studies, only discrepancies have resulted (18–20). Furthermore, none of them deeply investigated the underlying cellular and molecular mechanisms.

Considering these points, in the present study we used a series of genetic, biochemical, and pharmacological approaches to investigate the role of KYNPATH in the development of neuropathic pain. We demonstrate that neuropathic pain is abrogated when the KYNPATH is pharmacologically or genetically ablated. Mechanistically, peripheral nerve injury induces an accumulation of dendritic cells (DCs) that express IDO1 in the dorsal root leptomeninges (DRL), a process leading to an increase in the levels of Kyn in the spinal cord. In the spinal cord, Kyn is metabolized by astrocyte-expressed KMO into the pronociceptive metabolite 3-Hk. Ultimately, we also found that HAAO-derived QA is involved in neuropathic pain development via activation of glutamatergic NMDA receptors. These findings help elucidate the crucial role of DC-triggered KYNPATH in the development of neuropathic pain and also suggest targets for the development of drugs for neuropathic pain control.

## Results

### IDO1 mediates peripheral nerve injury–induced neuropathic pain.

**Figure 2:**
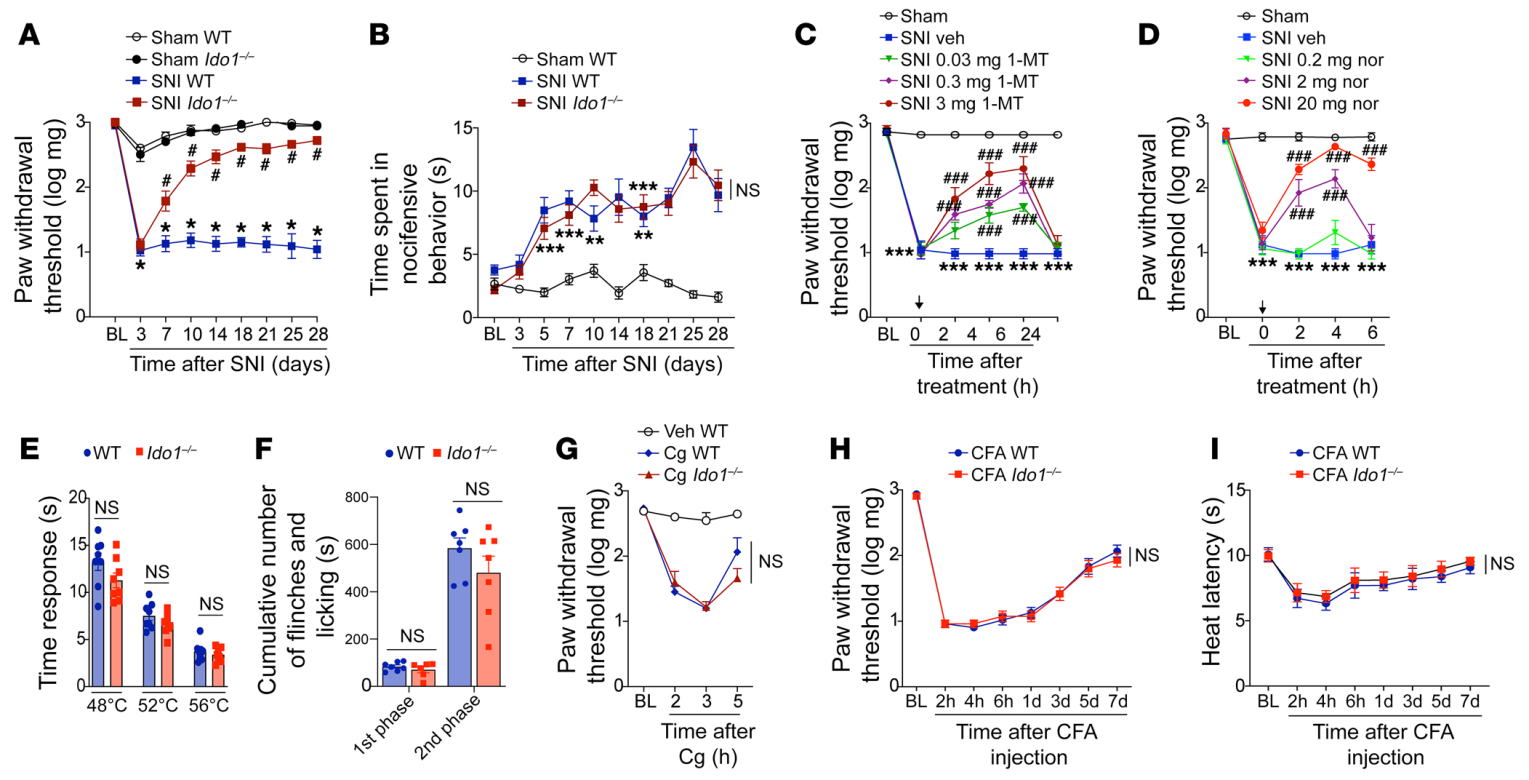
IDO1 is involved in the maintenance of neuropathic pain but has no role in nociceptive or inflammatory pain. (**A**) Mechanical and (**B**) cold nociceptive responses were evaluated before and up to 28 days after SNI and sham surgeries in WT and *Ido1^–/–^* mice. (*n* = 5–9). (**C** and **D**) Mechanical nociceptive threshold was determined before and 14 days after SNI. Mice were treated intraperitoneally (i.p.) with vehicle or 1-methyl-DL-tryptophan (1-MT, 0.03–3 mg/mouse) or norharmane (nor, 0.2–20 mg/kg), and mechanical allodynia was measured up to 24 hours after treatment (*n* = 5). (**E**) The nociceptive thermal threshold was tested in naive WT and *Ido1^–/–^* mice at 48°C, 52°C, and 56°C using the hot-plate test (*n* = 8). (**F**) Formalin (1%) was used to produce overt pain-like behavior. Total duration (seconds) of nociceptive behaviors for 0 to 10 minutes (1st phase) and for 10 to 50 minutes (2nd phase) after formalin injection was evaluated in WT and *Ido1^–/–^* mice (*n* = 7). (**G**) Mechanical nociceptive threshold using von Frey test (*n* = 4–5) was evaluated in WT and *Ido1^–/–^* mice followed by intraplantar injection of carrageenan (Cg, 100 μg per paw) or vehicle (saline). (**H** and **I**) Mechanical and thermal (heat) nociceptive threshold using von Frey test (*n* = 7) and Hargreaves’ test, respectively, were evaluated in WT and *Ido1^–/–^* mice followed by intraplantar injection of CFA (10 μL per paw). Data are expressed as mean ± SEM. *P < 0.05, **P < 0.01, ***P < 0.001 versus sham or vehicle; ^#^*P* < 0.05, ^###^*P* < 0.001 versus *Ido1^–/–^* mice by 2-way ANOVA with Bonferroni’s post hoc test (**A**–**D** and **G**–**I**) or unpaired, 2-tailed Student’s *t* test (**E** and **F**). NS, no statistical significance.

Peripheral nerve injury triggers activation of spinal cord glial cells, which participate in neuropathic pain development via the production of a vast range of pronociceptive mediators (21, 22). Given that KYNPATH, specially IDO1, is generally upregulated by inflammatory mediators (23), we first hypothesized that the neuroimmune response in the spinal cord after peripheral nerve injury could promote the upregulation of IDO1 and consequently the KYNPATH that in turn could participate in development of neuropathic pain. To test this initial hypothesis, we used a well-established model of peripheral nerve injury–induced neuropathic pain, the spared nerve injury (SNI) model, which is characterized by the development of mechanical and cold allodynia starting 3 days after nerve injury until experimental endpoints (day 21) in addition to a robust activation of microglial cells in the spinal cord when compared with sham-operated mice, as shown in Figure 1, B and C, and Supplemental Figure 1 (supplemental material available online with this article; https://doi.org/10.1172/JCI153805DS1) (24). The development of SNI-induced mechanical and cold allodynia (increase in pain hypersensitivity) was associated with an increase in mRNA and protein expression levels and activity of IDO1 in the ipsilateral dorsal horn of the spinal cord (Figure 1, D–G). To examine the involvement of IDO1 in neuropathic pain development, we first tested the phenotype of *Ido1*-null (*Ido1^–/–^*) mice in SNI-induced mechanical and cold allodynia. Notably, *Ido1^–/–^* mice developed mechanical allodynia that resembled that of wild-type (WT) mice in the early stage after SNI (3 days after surgery); however, mechanical allodynia in *Ido1^–/–^* mice began to ameliorate from days 7 to 28 after surgery (Figure 2A and Supplemental Figure 2, A and B). Notably, no difference was observed in SNI-induced cold allodynia between *Ido1^–/–^* and WT mice (Figure 2B). Second, WT mice were pharmacologically treated once at the peak of IDO1 expression (14 days after SNI) with 2 different IDO1 inhibitors: 1-methyl-tryptophan (1-MT) and norharmane (25). Corroborating the genetic data, both IDO1 inhibitors caused a transient and dose-dependent reduction in SNI-induced mechanical allodynia (Figure 2, C and D). Continuous high doses of 1-MT for 1 week (twice a day from days 14 to 20 after SNI) blocked mechanical allodynia over the treatment period (Supplemental Figure 3A). Importantly, mechanical allodynia returned to the same level as found in the SNI control group 2 days after the suspension of 1-MT treatment (Supplemental Figure 3A), indicating that IDO1 inhibition led to relief, but not resolution, of mechanical allodynia. Furthermore, a single treatment with 1-MT at 21 days after SNI induction also led to a reduction in mechanical allodynia (Supplemental Figure 3B). A potential role for the IDO1 in the activation of microglial cells in the spinal cord (ipsilateral dorsal horn) after peripheral nerve injury was ruled out since *Aif1* (IBA-1 gene) and *Cx3cr1* expression (marker of microglial cell activation) levels were comparable in WT and *Ido1^–/–^* mice over a time course after SNI (Supplemental Figure 4, A–C). We did not find significant changes in the marker of astrocyte activation, glial fibrillary acidic protein (*Gfap*), in the ipsilateral dorsal horn of the spinal cord after SNI even in the WT mice, a result that resembled the result with *Ido1^–/–^* mice (Supplemental Figure 4D). Of note, naive *Ido1^–/–^* mice exhibited similar thermal pain thresholds (over temperatures ranging from 48°C to 56°C) compared to WT mice (Figure 2E). Furthermore, compared to WT mice, naive *Ido1^–/–^* mice also showed no difference in either total nociceptive behavior in the first and second phases after formalin injection (Figure 2F) or carrageenan-induced mechanical inflammatory pain hypersensitivity (Figure 2G). In addition, complete Freund’s adjuvant–induced (CFA-induced) mechanical and thermal pain hypersensitivity in *Ido1^–/–^* mice did not differ from those in WT mice (Figure 2, H and I). Collectively, these data indicate that IDO1 is involved in the development of neuropathic pain but has no role in nociceptive (physiological) or inflammatory pain. Furthermore, these results also indicate that IDO1-derived kynurenines have no role in microglial cell activation in the dorsal horn of the spinal cord after peripheral nerve injury.

### IDO1 in hematopoietic cells mediates neuropathic pain.

**Figure 3:**
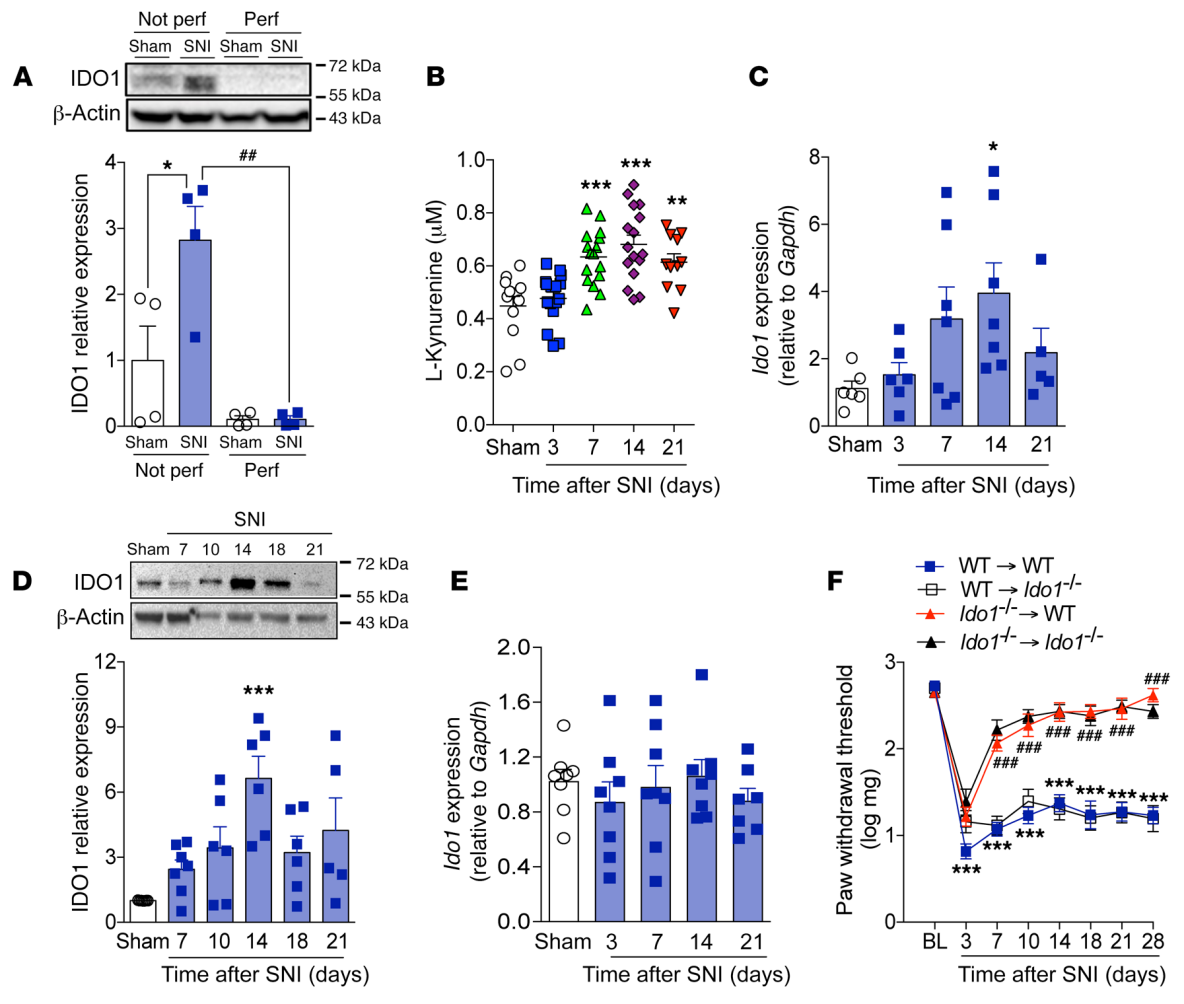
Upregulation of IDO1 in peripheral hematopoietic cell compartment mediates neuropathic pain. (**A**) Western blotting analysis of IDO1 expression in the dorsal horn of the spinal cord with or without transcardiac perfusion with PBS after sham or SNI surgery (*n* = 4). (**B**) Time course of kynurenine levels in the serum of mice after sham (14 days) and SNI surgeries (*n* = 11–17 per time point). Time course of *Ido1* mRNA (**C**) (*n* = 5–7 per time point) and protein (**D**) (*n* = 7 per time point) expression in the draining lymph nodes after sham or SNI surgery. (**E**) Time course of *Ido1* mRNA expression in spleen after sham (14 days) or SNI surgeries (*n* = 7–8 per time point). (**F**) Mechanical nociceptive threshold of WT→WT, *Ido1^–/–^*→*Ido1^–/–^*, *Ido1^–/–^*→WT, and WT→*Ido1^–/–^* chimeric mice before (baseline, BL) and up to 28 days after SNI (*n* = 7). Data are expressed as mean ± SEM. *P < 0.05, **P < 0.01, ***P < 0.001 versus sham; ^##^*P* < 0.01, ^###^*P* < 0.001 versus *Ido1^–/–^* mice or perfused group by 1-way ANOVA with Bonferroni’s post hoc test (**A**–**E**) or 2-way ANOVA with Bonferroni’s post hoc test (**F**).

Despite indications of IDO1 protein expression in spinal cord based on Western blotting data (Figure 1E), no significant immunostaining for IDO1 was observed in the spinal cord after SNI (data not shown). These finding led us to hypothesize that IDO1 is not upregulated in the spinal cord parenchyma but could be located on circulating blood cells. To test this hypothesis, we designed one set of experiments to collect spinal cord tissue with/without transcardiac perfusion (PBS) before sample collection. Perfusing mice before harvesting the spinal cord led to elimination of IDO1 protein expression in the spinal cord tissue, as assessed by Western blotting (Figure 3A). This finding suggests that IDO1, which plays a role in the development of neuropathic pain, is not expressed (upregulated) in resident cells of the spinal cord; however, it is within the vasculature and in the circulating (blood) immune cells. In line with this hypothesis, systemic plasma levels of Kyn increased after SNI and peaked 14 days after peripheral nerve injury (Figure 3B). Corroborating the idea that IDO1 is upregulated in leukocytes after peripheral nerve injury, IDO1 expression (mRNA and protein) was found to increase in the draining lymph nodes (dLNs), corresponding to nerve injury (popliteal and inguinal) as shown in Figure 3, C and D, but not in the spleen after SNI surgery (Figure 3E). To test whether IDO1 expression in immune cells is important for the development of neuropathic pain, bone marrow (BM) chimeric mice were generated. Irradiated *Ido1^–/–^* mice that received BM from *Ido1^–/–^* mice were still resistant to SNI-induced mechanical allodynia when compared with irradiated WT mice that received BM from WT mice (Figure 3F). Adoptive transfer of BM from WT mice to irradiated *Ido1^–/–^* mice restored mechanical allodynia that resembled irradiated WT mice that received WT BM (Figure 3F). On the other hand, irradiated WT mice receiving BM from *Ido1^–/–^* mice became resistant to SNI-induced mechanical allodynia (Figure 3F). These results indicate that IDO1 expressed by peripheral hematopoietic cells is essential for neuropathic pain development after peripheral nerve injury.

### DC-expressed IDO1 mediates neuropathic pain.

**Figure 4:**
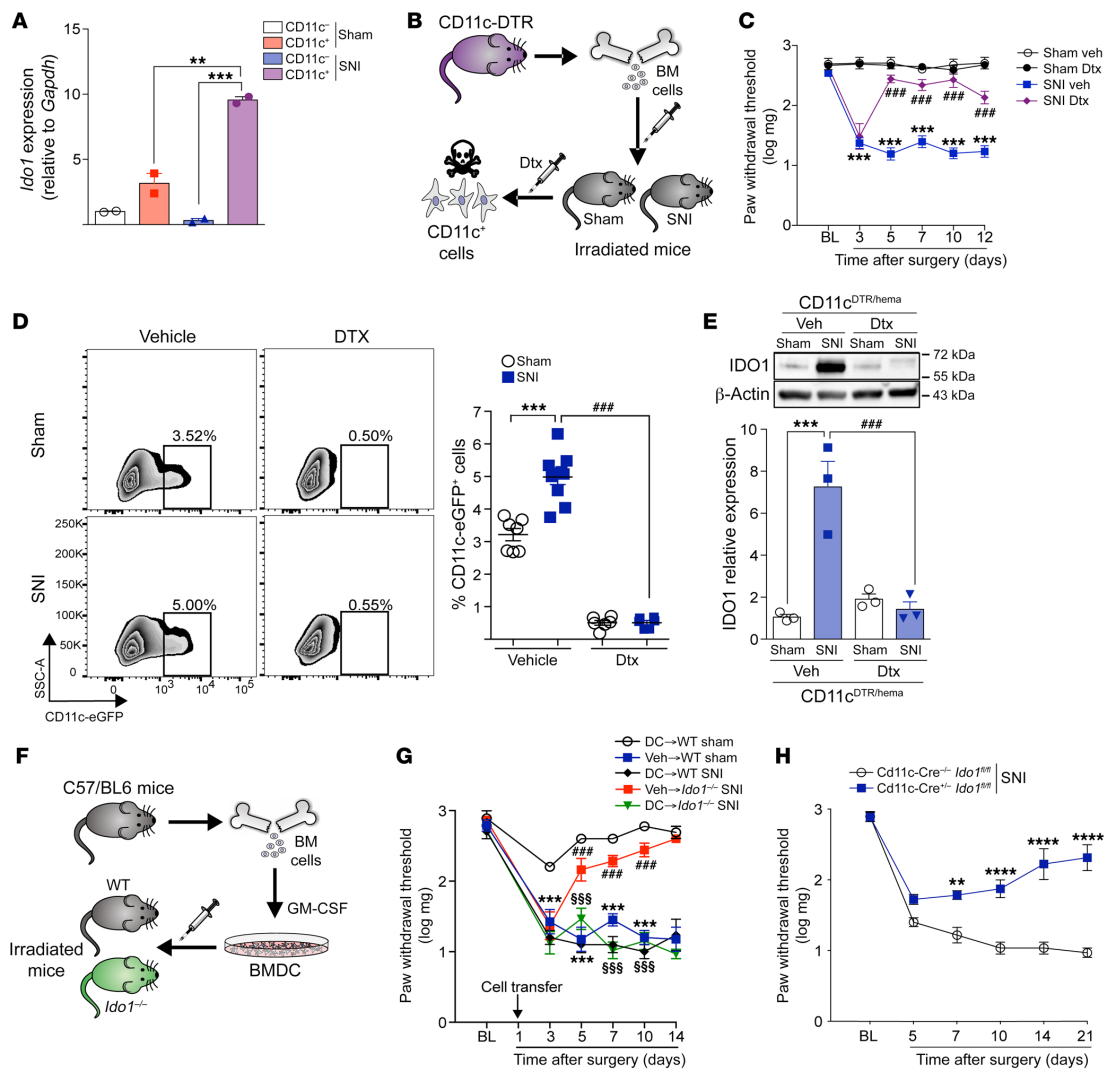
DC-expressed IDO1 contributes to the maintenance of neuropathic pain. (**A**) *Ido1* mRNA expression in CD11c^+^ or CD11c^–^ cells isolated from the draining lymph nodes 14 days after sham or SNI surgery (*n* = 2 pooled from 5). (**B**) Representative scheme of chimeric CD11c^DTR/hema^ mice establishment. (**C**) Mechanical nociceptive threshold was determined before and up to 12 days after sham or SNI surgery in chimeric CD11c^DTR/hema^ mice treated with vehicle or diphtheria toxin (Dtx; 16 ng Dtx/g, i.p.) (*n* = 6). (**D**) Representative dot plots and quantification (percentage) of CD11c^+^ DCs in the draining lymph nodes harvested 14 days after sham or SNI surgery from chimeric CD11c^DTR/hema^ mice treated with vehicle or Dtx (*n* = 6–10). (**E**) Western blotting analysis of IDO1 expression in the draining lymph nodes harvested 14 days after sham or SNI surgery from chimeric CD11c^DTR/hema^ mice treated with vehicle or Dtx (*n* = 3). (**F**) Representative scheme of DC differentiation and transfer to *Ido1^–/–^* or WT mice. (**G**) Mechanical nociceptive threshold before (BL) and up to 14 days after sham or SNI in WT and *Ido1^–/–^* mice that received in vitro–differentiated DCs 1 day after surgeries (*n* = 5–6). (**H**) Mechanical nociceptive threshold was evaluated before and up to 21 days after SNI surgery in mice conditionally lacking *Ido1* in CD11c^+^ cells (*CD11c*-*Cre^+/–^* *Ido1^fl/fl^* mice) or control littermates (*CD11c*-*Cre^–/–^* *Ido1^fl/fl^*) (*n* = 4–6). Data are expressed as mean ± SEM. **P < 0.01, ***P < 0.001, ****P < 0.0001 versus sham littermate control; ^###^*P* < 0.001 versus WT mice or treatment; ^§§§^*P* < 0.001 versus BM *Ido1^–/–^* by 1-way ANOVA with Bonferroni’s post hoc test (**A**, **D**, and **E**) or 2-way ANOVA with Bonferroni’s post hoc test (**C**, **G**, and **H**).

Next, we sought to identify IDO1-expressing immune cells that contribute to neuropathic pain development. We and others have identified DCs as the main immune cell population expressing IDO1 in many pathological conditions such as cancer, leukemia, arthritis, and viral and bacterial infections (26–29). Thus, we hypothesized that DCs would be the cellular source of IDO1 that mediates neuropathic pain. To test this hypothesis, several approaches were used. First, SNI was induced in CD11c^Yfp^ mice, and CD11c^+^ cells (DCs) were isolated from dLNs by fluorescence-activated cell sorting (FACS) as shown in Supplemental Figure 5. Of note, it was found that *Ido1* mRNA expression was higher in CD11c^+^ cells when compared with CD11c^–^ cells in sham-operated mice (Figure 4A). Moreover, SNI-induced upregulation of *Ido1* was only observed in CD11c^+^, but not in CD11c^–^, cells (Figure 4A). Corroborating these data, immunofluorescence analyses of the dLNs revealed that IDO1 was mainly expressed in CD11c^+^ cells (Supplemental Figure 6). To further address the importance of DCs for neuropathic pain development, transgenic mice expressing the diphtheria toxin (Dtx) receptor (DTR) under the control of the CD11c (*Itgax*) promoter (CD11c-DTR-eGFP mice) were used to allow conditional depletion of DCs. As CD11c-DTR mice are sensitive to Dtx due to adverse effects causing fatal fulminant myocarditis (30), we reconstituted lethally irradiated WT mice with BM cells from CD11c-DTR mice (donor) to generate mice that present DTR only in CD11c^+^ cells of the hematopoietic compartment (CD11c^DTR/hema^ mice) (Figure 4B). Following reconstitution, CD11c^+^ DCs can be depleted in these mice by Dtx administration (31). No adverse effects are associated with the chronic administration of Dtx to CD11c^DTR/hema^ chimeric mice (31). Two months after BM reconstitution, SNI or sham surgery was performed in CD11c^DTR/hema^ mice followed by treatment with Dtx or vehicle. Conditional depletion of DCs in CD11c^DTR/hema^ by Dtx treatment caused a significant reduction in mechanical allodynia after SNI (Figure 4C). Flow cytometry analysis of dLNs confirmed that Dtx treatment caused depletion of CD11c^+^ DCs (Figure 4D). Of note, Dtx treatment had no effect on the mechanical threshold or mechanical allodynia in WT mice after either sham or SNI surgery (Supplemental Figure 7). The population of CD11c^+^ DCs in dLNs increased after SNI compared with sham-operated mice (Figure 4D), suggesting that nerve injury per se incited greater influx of DCs into dLNs. Additionally, SNI-induced IDO1 upregulation was not observed in dLNs of DC-depleted mice (Figure 4E). To further confirm that DCs expressing IDO1 are important for the neuropathic pain development caused by peripheral nerve injury, in vitro–differentiated BMDCs were generated and transferred as shown in Figure 4F (31). Whereas *Ido1^–/–^* mice are resistant to SNI-induced mechanical allodynia, *Ido1^–/–^* mice that received WT BMDCs became susceptible (Figure 4G). Finally, conditional mice lacking *Ido1* expression in CD11c^+^ cells (*CD11c*-*Cre*^+/–^ *Ido1^fl/fl^* mice) were also found to be resistant to development of mechanical allodynia after SNI compared with littermate controls (*CD11c*-*Cre^–/–^* *Ido1^fl/fl^* mice), as shown in Figure 4H and Supplemental Figure 8, A and B. Collectively, these results indicate that IDO1 expressed mainly by DCs plays an important role in the development of neuropathic pain after peripheral nerve injury.

### DC-expressed IDO1 in DRL mediates neuropathic pain.

**Figure 5:**
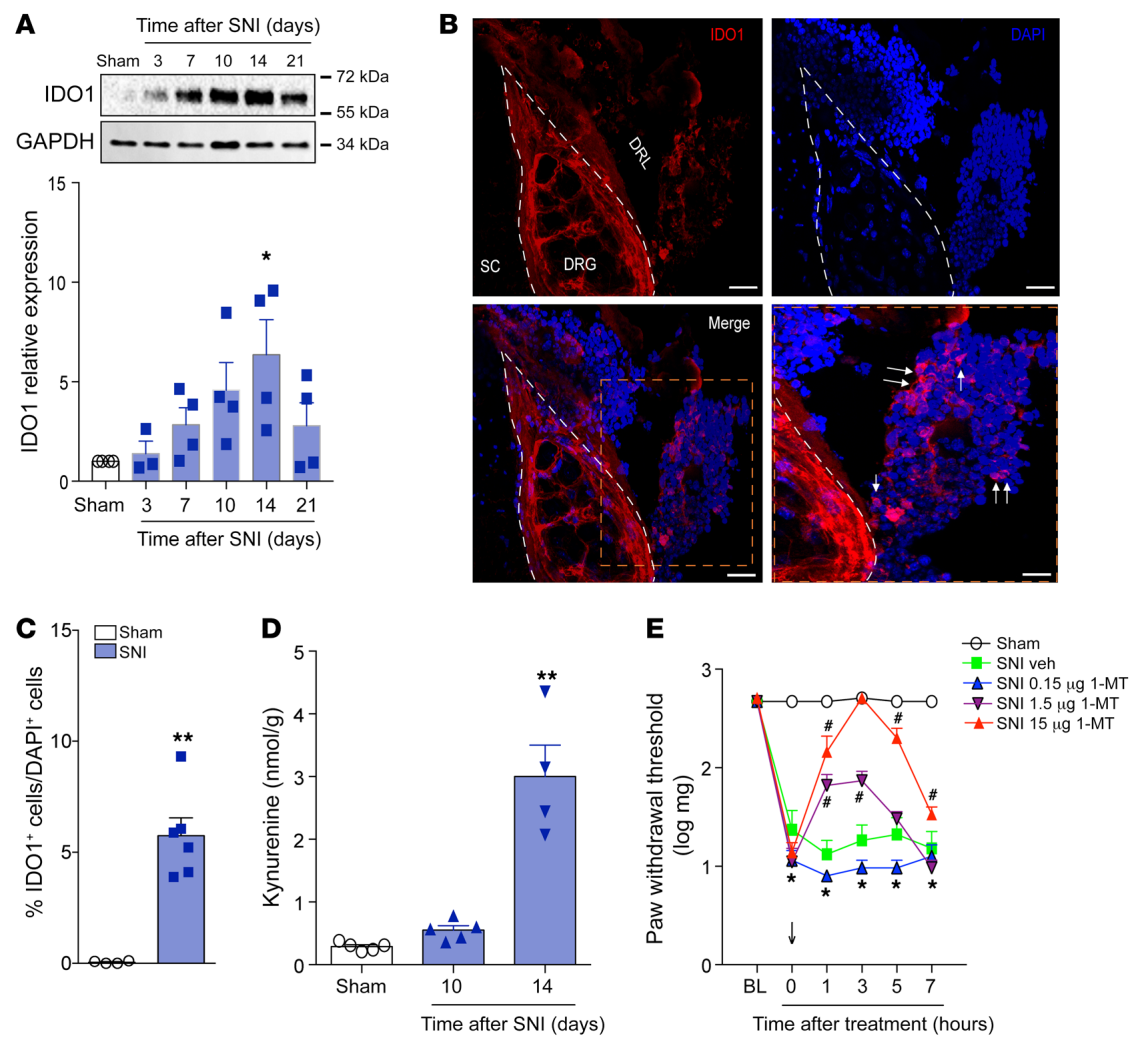
Cells expressing IDO1 accumulate in the DRL after SNI and contribute to the maintenance of neuropathic pain. (**A**) Time course of IDO1 expression in the DRGs plus DRL tissues (*n* = 4 per time point). (**B**) Representative images showing immunoreactivity for IDO1 (red color) double labeled with DAPI (cell nuclei, blue) in the ipsilateral region containing DRG (L4), DRL, and spinal cord (SC) from SNI mice (14 days after SNI). Scale bars: 100 μm. (**C**) Quantification of IDO1-expressing cells in the DRL from SNI mice (14 days after SNI) or sham mice (*n* = 6). (**D**) Time course of kynurenine levels in the ipsilateral dorsal horn of the spinal cord of mice after sham (14 days) and SNI surgeries (*n* = 5 per time point). (**E**) Mechanical nociceptive threshold was determined before and 14 days after SNI. Mice were treated intrathecally with vehicle or 1-methyl-DL-tryptophan (1-MT, 0.15–15 μg/site) and mechanical allodynia was measured up to 7 hours after treatment (*n* = 5). Data are expressed as mean ± SEM. *P < 0.05, **P < 0.001 versus sham group; ^#^*P* < 0.05 versus vehicle-treated mice by 1-way ANOVA with Bonferroni’s post hoc test (**A** and **D**), unpaired 2-tailed Student’s *t* test (**C**), or 2-way ANOVA with Bonferroni’s post hoc test (**E**).

Evidence can be found in the literature describing that peripheral immune cells (such as T cells) fail to infiltrate the spinal cord or dorsal root ganglion (DRG) after peripheral nerve injury, but they might infiltrate/accumulate in the DRL of the somatosensory pathways (32–35). These DRL-infiltrating leukocytes, especially CD4^+^ αβ T cells, play a role in the development of neuropathic pain (32). Therefore, we next sought to investigate whether DCs expressing IDO1 would also be capable of infiltrating/accumulating in the DRL. Initially, we harvested DRGs (L3–L5) together with the corresponding DRL and analyzed the expression of IDO1 after SNI induction. Western blotting analyses showed that the expression of IDO1 was upregulated in these tissues and peaked at 14 days after SNI induction (Figure 5A). We then assessed IDO1 localization in these tissues. IDO1-expressing cells were found to increase in the vicinity of DRGs, an area that was assumed to be the DRL, but no obvious IDO1-expressing cells in the parenchyma of L4–L5 DRGs were found (Figure 5, B and C). Since IDO1-expressing cells were reported in the DRL after SNI and leukocytes in the DRL might influence spinal cord processes (32), we sought to analyze whether IDO1’s downstream metabolite (Kyn) would also have increased in the spinal cord. Mass spectrometry analyses of the dorsal horn of spinal cord tissue homogenate revealed an increase in the levels of Kyn in a time- and IDO1-dependent manner (Figure 5D and Supplemental Figure 9A). Notably, Kyn levels in the spinal cord peaked 14 days after SNI, which corresponded to the peak of IDO1 expression in the DRLs (Figure 5D). To investigate whether IDO1 expressed in accumulated cells in the DRL might participate in the development of neuropathic pain, SNI animals (14 days after injury) were intrathecally treated with the IDO1 inhibitor 1-MT. Notably, SNI-induced mechanical allodynia was inhibited by intrathecal treatment with 1-MT in a dose-dependent manner (Figure 5E). In addition, intrathecal treatment of SNI animals with 1-MT also led to a reduction in the levels of Kyn in the spinal cord (Supplemental Figure 9B), indicating that IDO1-expressing cells in the DRL are probably the source of this KYNPATH metabolite that was detected in the spinal cord.

**Figure 6:**
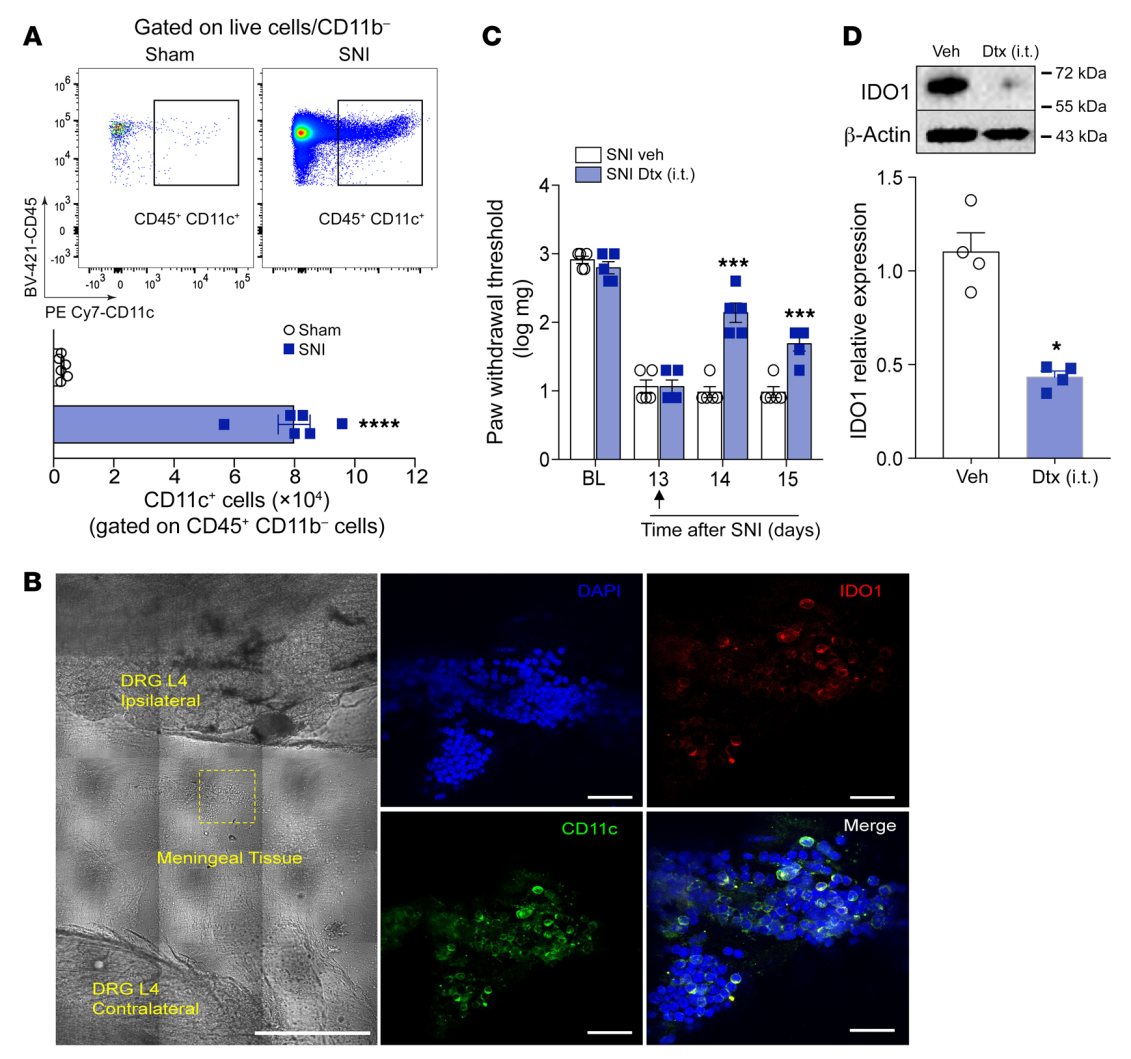
IDO1-expressing DCs accumulate in the DRL after SNI and contribute to the maintenance of neuropathic pain. (**A**) Representative dot plots and quantification of CD45^+^CD11b^–^CD11c^+^ cells (DCs) in DRGs plus DRLs harvested 14 days after sham or SNI surgery from WT mice (*n* = 5). (**B**) Representative images showing immunoreactivity for IDO1 (red color) double labeled with anti-CD11c (DCs) in the ipsilateral DRLs (L4) from SNI mice (14 days after SNI). Scale bars: 50 μm (left) and 100 μm (right). (**C**) Mechanical nociceptive threshold was determined before and 13 days after SNI. Mice were treated intrathecally with vehicle or diphtheria toxin (Dtx, 20 ng/site) and mechanical allodynia was measured up to 48 hours after treatment (*n* = 5). (**D**) Western blotting analysis of IDO1 expression in the DRGs plus DRL tissues harvested 15 days after SNI mice were treated with vehicle or Dtx (*n* = 4). Data are expressed as mean ± SEM. *P < 0.05, ***P < 0.001, ****P < 0.0001 versus sham group by unpaired 2-tailed Student’s *t* test (**A** and **D**) or 1-way ANOVA with Bonferroni’s post hoc test (**C**).

We then wanted to evaluate the cell subtype that expresses IDO1 in the DRL after peripheral nerve injury. Based on our previous data, DCs that expressed IDO1 were immediately included in our hypothesis. Initially, flow cytometry analyses revealed that an increase in DCs (CD45^+^CD11c^+^CD11b^–^ cells) also occurred in the DRL, 14 days after SNI induction (Figure 6A and Supplemental Figure 10, A and B). Remarkably, IDO1-expressing cells that accumulated in the DRL after SNI induction also expressed CD11c (Figure 6B and Supplemental Figure 11A). Notably, IDO1-expressing cells in the DRL did not express a classical marker of macrophages (IBA-1; Supplemental Figure 11B). These results appear to confirm that IDO1-expressing CD11c^+^ cells that accumulated in DRLs after SNI were indeed DCs. In an attempt to eliminate and confirm the role of CD11c^+^ cells expressing IDO1 in the DRLs involved in the development of neuropathic pain, an SNI model was induced in CD11c-DTR mice. After 13 days, these mice were intrathecally treated with Dtx or vehicle, and mechanical allodynia was determined followed by the examination of IDO1 expression. We found that this treatment caused a reduction in SNI-induced mechanical allodynia (Figure 6C), which was associated with a reduction in IDO1 expression in DRLs (Figure 6D). Collectively, these results indicate that IDO1-expressing DCs accumulate in the DRLs and mediate the development of neuropathic pain.

### Spinal astrocytes expressing KMO mediate neuropathic pain.

**Figure 7:**
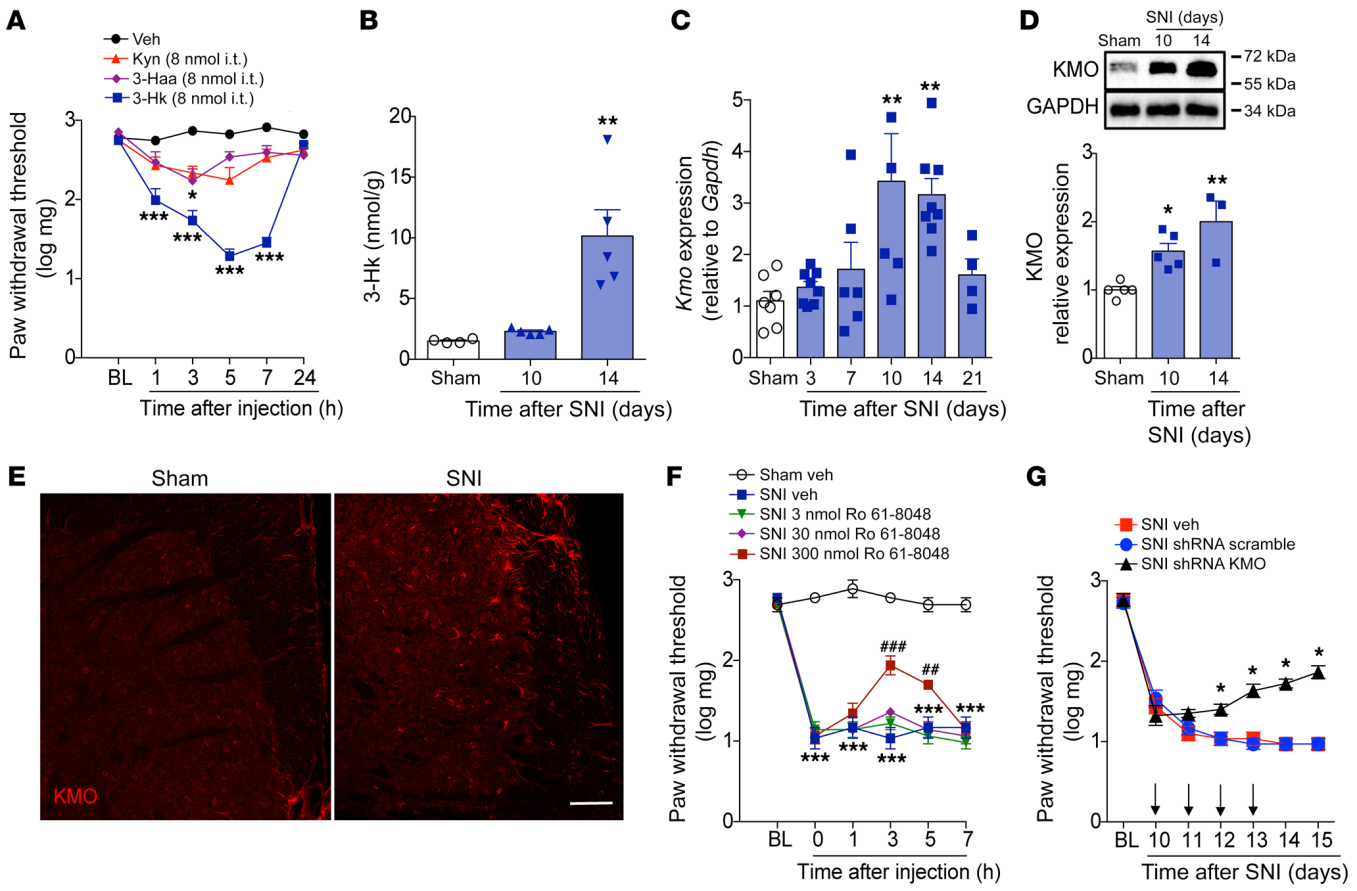
KMO is upregulated in the spinal cord after SNI and mediates neuropathic pain. (**A**) Mechanical nociceptive threshold was evaluated before and up to 24 hours after intrathecal injection of equimolar doses (8 nmol) of kynurenine (Kyn), 3-hydroxykynurenine (3-Hk), 3-hydroxyanthranilic acid (3-Haa), or vehicle (saline) in WT mice (*n* = 5–6). This panel shows representative curves obtained in the dose-response analysis of mechanical allodynia caused by equimolar doses of Kyn, 3-Hk, 3-Haa in WT mice (see Supplemental Figure 8). (**B**) Time course of 3-Hk levels in the ipsilateral dorsal horn of the spinal cord of mice after sham (14 days) and SNI surgeries (*n* = 4–5 per time point). (**C**) Time course of *Kmo* mRNA expression in the ipsilateral dorsal horn of the spinal cord spinal cord after sham (14 days) and SNI surgeries. (*n* = 5–8). (**D**) Western blotting analysis of KMO expression in the ipsilateral dorsal horn of the spinal cord after sham (14 days) or SNI surgery (10 and 14 days) (*n* = 4–5). (**E**) Representative image of KMO expression analyzed by immunofluorescence in the dorsal horn of the spinal cord after sham or SNI induction (14 days). Scale bar: 50 μm. (**F**) Mechanical nociceptive threshold was determined before and 14 days after SNI. Mice were then treated intrathecally (i.t.) with Ro 61-8048 (KMO inhibitor; 3–300 nmol) or vehicle and mechanical allodynia was measured up to 7 hours after treatments (*n* = 5). (**G**) Mechanical nociceptive threshold was determined before and 10 days after SNI followed by intrathecal treatment with shRNA against KMO, shRNA scramble, or vehicle (indicated arrows) and mechanical allodynia was measured up to 15 days after SNI (*n* = 6). Data are expressed as mean ± SEM. *P < 0.05, **P < 0.01, ***P < 0.001 versus sham or saline injected; ^##^*P* < 0.01, ^###^*P* < 0.001 versus mice treated with Ro 61-8048 or scramble shRNA by 2-way ANOVA with Bonferroni’s post hoc test (**A**, **F**, and **G**) or 1-way ANOVA with Bonferroni’s post hoc test (**B**–**D**).

Next, we investigated the mechanisms by which IDO1 activity in the DRL mediates neuropathic pain. As we previously showed, the accumulation of DCs expressing IDO1 in the DRLs led to an increase in Kyn levels in the spinal cord (Figure 5D) after peripheral nerve injury. We then investigated whether this spinal cord Kyn would be directly involved in the development of neuropathic pain. In addition, as Kyn in the CNS is rapidly converted into downstream metabolites (36), we also hypothesized that the Kyn that reached the spinal cord after peripheral nerve injury could be converted into downstream neuroactive metabolites (such as 3-Hk and 3-Haa), and these metabolites could directly account for neuropathic pain. To gain further information about these possibilities, we initially tested the capability of Kyn and its immediate downstream metabolites, 3-Hk and 3-Haa, to produce mechanical pain hypersensitivity when injected intrathecally into naive animals. When equimolar doses of these molecules were injected, it was found that only 3-Hk produced robust mechanical pain hypersensitivity (Figure 7A and Supplemental Figure 12, A–C).

The results showing that 3-Hk possesses more potent pronociceptive activity than Kyn led us to consider that IDO1 downstream metabolites other that Kyn (such as 3-Hk) would be more important for neuropathic pain development. Because KMO is the rate-limiting downstream enzyme in the KYNPATH that oxidatively metabolizes Kyn into 3-Hk (4), we then hypothesized that the Kyn that reaches the spinal cord might be metabolized by KMO to generate downstream pronociceptive factors, such as 3-Hk, which might be involved in neuropathic pain development. Consistent with this hypothesis, levels of the KMO downstream metabolite 3-Hk increased in the ipsilateral dorsal horn of the spinal cord after SNI induction (Figure 7B), a process that was dependent on IDO1 activity in the DRLs (Supplemental Figure 9C). This event was also associated with an increase in KMO expression (mRNA and protein) in the ipsilateral dorsal horn of the spinal cord after SNI induction (Figure 7, C–E). Notably, no significant changes in the levels of Kyna were observed in the spinal cord after SNI, although the 3-Hk/Kyna ratio increased significantly (Supplemental Figure 13, A and B). Additionally, we found that pharmacological treatment (intrathecally) with the KMO inhibitor Ro 61-8048 (37) also led to a reduction in the SNI-induced mechanical allodynia in a dose-dependent manner (Figure 7F). Furthermore, intrathecal treatment with shRNA against KMO also caused a reduction in SNI-induced mechanical allodynia (Figure 7G).

**Figure 8:**
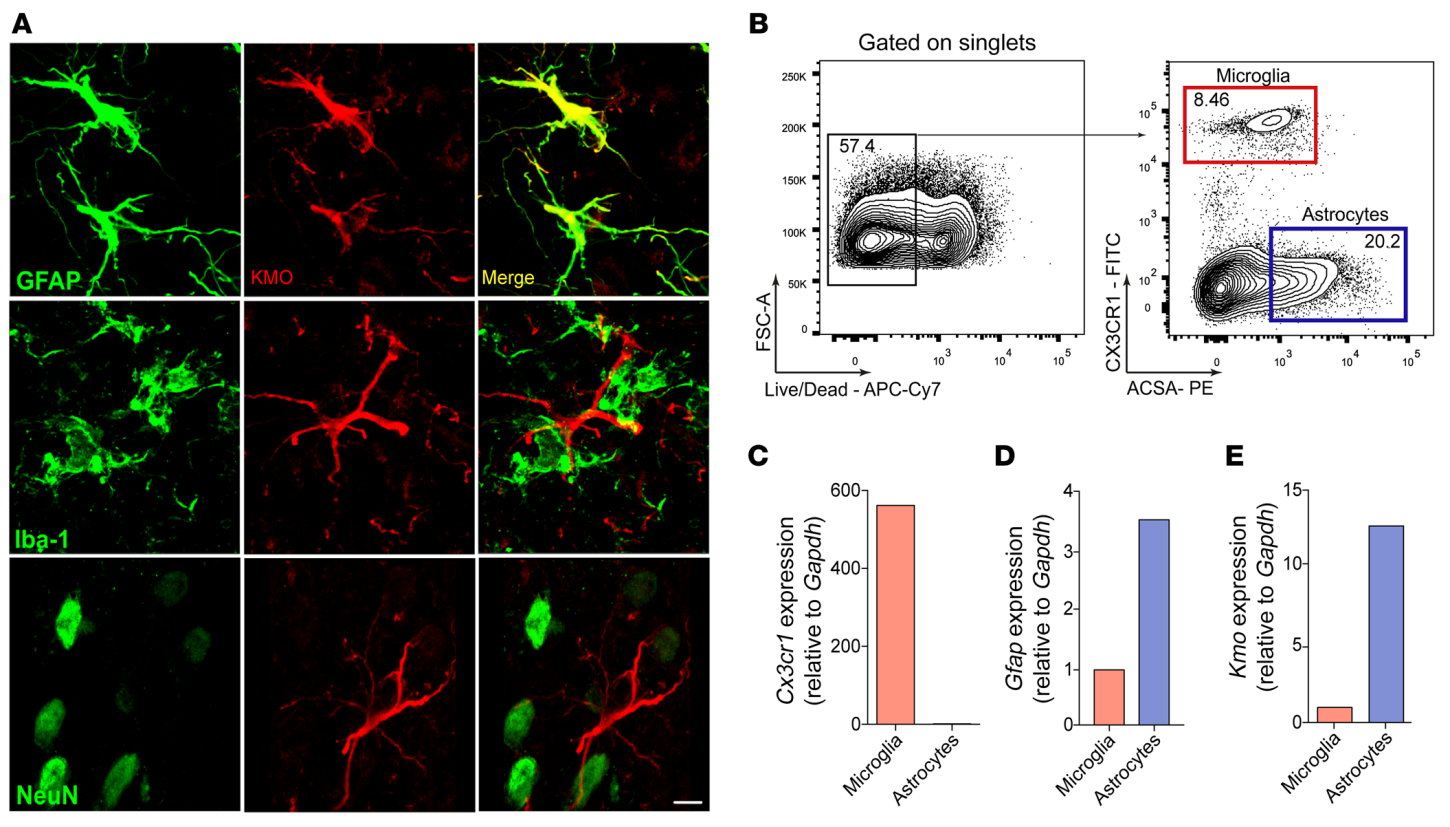
KMO expression cell profile in the spinal cord after SNI. (**A**) Representative images showing immunoreactivity for KMO (red color) double labeled with anti-GFAP (astrocytes), anti–IBA-1 (microglia), or anti-NeuN (neurons) in the ipsilateral dorsal horn of the spinal cord from SNI mice (14 days after SNI). Scale bar: 5 μm. (**B**) Representative FACS strategy for isolating microglial cells (CX3CR1^–^eGFP^+^ cells) and astrocytes (ACSA2^+^) from spinal cord. (**C**–**E**) *Cx3cr1*, *Gfap*, and *Kmo* mRNA expression in microglia and astrocytes isolated from the spinal cord (pooled from 8).

**Figure 9:**
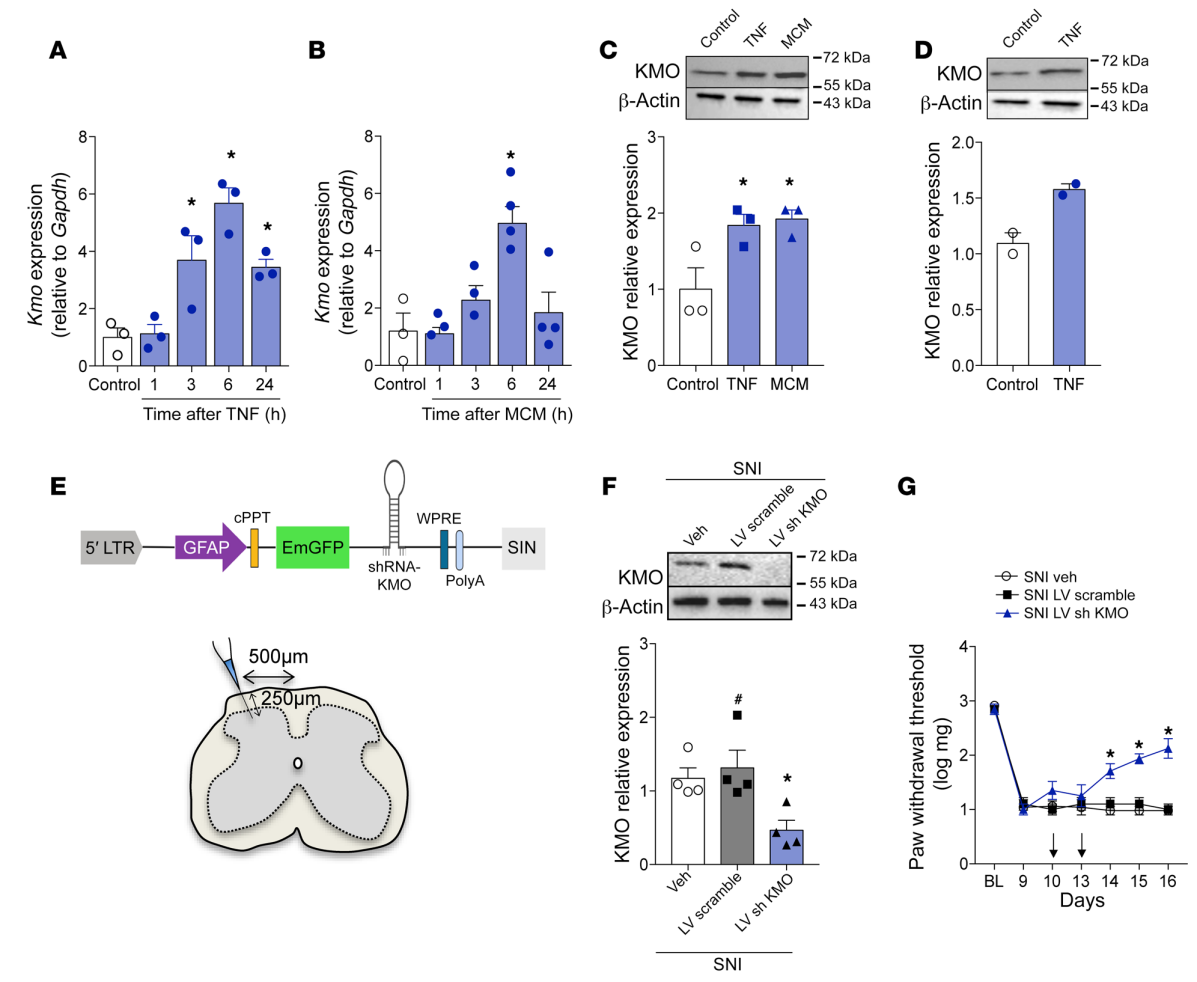
Astrocyte-expressed KMO maintains neuropathic pain. Primary cultured astrocytes from mouse cortex were stimulated with (**A**) TNF (10 ng/mL) or (**B**) microglia-conditioned medium (MCM). After indicated time points, mRNA was extracted and *Kmo* expression was analyzed by real-time PCR (*n* = 3–4). (**C**) Primary cultured astrocytes from mouse cortex or (**D**) differentiated U87-MG cells were stimulated with TNF (10 ng/mL) or MCM (*n* = 2–3). The expression of KMO in the cell extract was evaluated 24 hours after stimulation by Western blotting. (**E**) Schematic of the astrocyte-specific shRNA lentiviral vector (LV) used to knock down KMO in astrocytes of the spinal cord. (**F**) Mechanical nociceptive threshold was determined before and 9 days after SNI followed by intrathecal treatment with lentiviral vectors expressing shRNA control or shRNA Kmo (*n* = 4–5) on days 10 and 13 after SNI. (**G**) Mechanical allodynia was measured up to 16 days after SNI and ipsilateral dorsal horn of the spinal cord was collected for analyses of KMO expression (*n* = 4). Data are expressed as mean ± SEM. *P < 0.05 versus medium treated; ^#^*P* < 0.05 versus mice treated with scramble shRNA by 1-way ANOVA with Bonferroni’s post hoc test (**A**–**C** and **G**) or 2-way ANOVA with Bonferroni’s post hoc test (**F**).

To characterize which spinal cord cell subtype might be expressing KMO and would be important for 3-Hk production, a series of experiments were performed. First, immunofluorescence of spinal cord slices from SNI animals revealed that KMO expression was detected exclusively in GFAP^+^ cells but not in IBA-1^+^ microglia or NeuN^+^ neurons (Figure 8A, Supplemental Figure 14, and Supplemental Videos 1–3), indicating selective KMO expression by astrocytes, which are in close contact with spinal cord neurons (Supplemental Video 3). Next, we performed cell sorting analyses of microglia and astrocytes from spinal cord (38, 39) followed by the analyses of *Cx3cr1*, *Gfap*, and *Kmo* expression levels (Figure 8, C–E). In support of the immunofluorescence data, we found that *Kmo* mRNA expression was higher in astrocytes than in microglial cells (Figure 8E). To further support these data, pure primary astrocytes from newborn mouse cortex (Supplemental Figure 15) were cultured, after which the expression of KMO was analyzed. Activation of primary culture astrocytes with tumor necrosis factor (TNF) or microglia-conditioned medium (MCM) induced upregulation in the expression (mRNA and protein) of KMO (Figure 9, A–C). Importantly, activation of differentiated U87-MG, an immortalized human astrocytic cell line (40), induced an increase in the expression of KMO (Figure 9D). Finally, we sought to confirm whether KMO expressed in spinal cord astrocytes is important for the development of neuropathic pain. To examine that association, we knocked down the expression of KMO specifically in astrocytes using a lentivirus-delivered shRNA (sh KMO) expressed under the control of the *Gfap* promoter, as shown in Figure 9E (41, 42). The lentivirus was administered intraspinally (43) 10 and 13 days after SNI induction, and a lentivirus carrying a nontargeting shRNA was used as a control (sh Scramble). KMO silencing in spinal cord astrocytes (Figure 9F) led to a reduction in SNI-induced mechanical allodynia (Figure 9G). Collectively, these results indicate that, after peripheral nerve injury, astrocytes expressing KMO mediate neuropathic pain development probably through conversion of Kyn into downstream pronociceptive factors, such as 3-Hk.

### HAAO-derived QA also plays a role in the maintenance of neuropathic pain.

**Figure 10:**
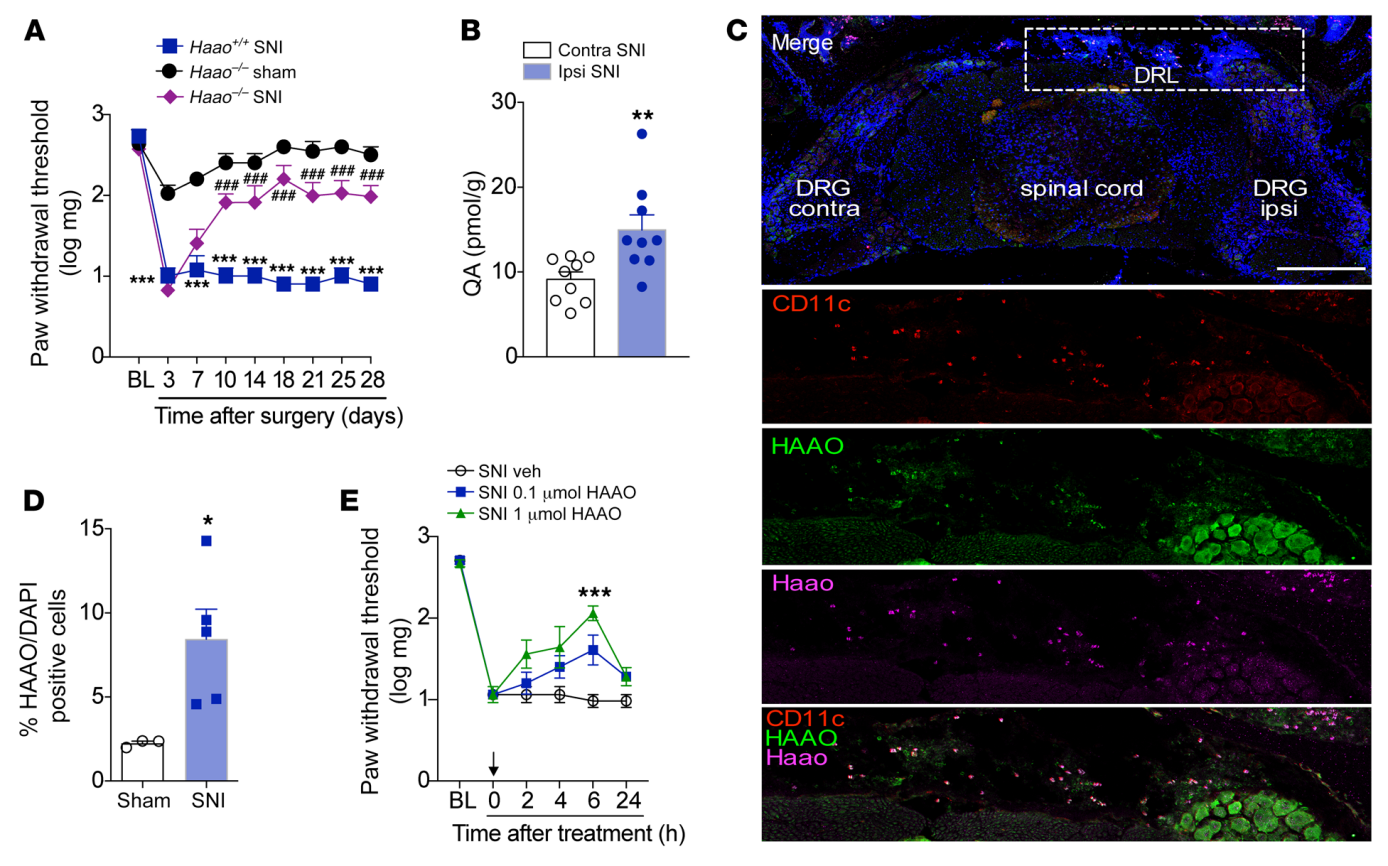
HAAO-derived QA mediates neuropathic pain through the upmodulation of NMDA currents. (**A**) Mechanical nociceptive threshold was evaluated before and up to 28 days after SNI and sham surgeries in 3-hydroxyanthranilic acid dioxygenase–knockout (*Haao^–/–^*) and *Haao^+/+^* mice (*n* = 4–8). (**B**) Levels of quinolinic acid (QA) were determined in the contralateral and ipsilateral dorsal horn of the spinal cord of mice after SNI surgery (14 days after surgery; *n* = 9). (**C**) Representative images of in situ hybridization (RNAscope) analysis of *Haao* (magenta) triple stained for HAAO (green) and CD11c (red) immunoreactivity and DAPI (cell nuclei, blue) in the ipsilateral region containing DRG (L4), DRL, and spinal cord from mice harvested 14 days after SNI surgery. Scale bar: 128 μm. (**D**) Quantification of *Haao* and HAAO-expressing cells in the DRL from SNI mice (14 days after SNI) or sham mice (*n* = 3–5). (**E**) Mechanical nociceptive threshold was determined before and 14 days after SNI. Mice were treated intrathecally with vehicle or HAAO inhibitor (0.1 and 1 μmol) and mechanical allodynia was measured up to 24 hours after treatment (*n* = 6). Data are expressed as mean ± SEM. *P < 0.05, **P < 0.01, ***P < 0.001 versus sham or vehicle; ^###^*P* < 0.001 versus *Haao^–/–^* mice by 2-way ANOVA with Bonferroni’s post hoc test (**A** and **E**) or unpaired 2-tailed Student’s *t* test (**B** and **D**).

The final step in the KYNPATH is HAAO-mediated generation of QA (44). Based on this step, we tested whether HAAO-dependent production of QA might also be involved in the development of neuropathic pain. SNI-induced acute (earlier time points) mechanical allodynia developed in *Haao^–/–^* mice in a manner resembling their WT littermates, but unlike WT mice, *Haao^–/–^* mice became resistant to mechanical allodynia after this initial period (Figure 10A). Supporting a role for an HAAO/QA axis in the development of neuropathic pain, we found a significant increase in QA levels in the dorsal horn of the spinal cord ipsilateral to the SNI surgery (Figure 10B). Next, we sought to identify the cellular source of HAAO in the nociceptive pathway. For that purpose, we took advantage of the whole preparation containing the lumbar spinal cord together with, DRLs, and DRGs (L3–L5), as shown in Figure 10C. With this preparation, it was possible to observe a massive increase in cells on the ipsilateral side of the DRL related to SNI surgery (Figure 10C). Notably, the analyses of mRNA using in situ hybridization (RNAscope) revealed that these DRL-accumulating cells express *Haao* (Figure 10, C and D). Immunostaining also revealed the HAAO protein in the DRL areas of the same cells (Figure 10C). Additionally, these cells expressing *Haao*/HAAO in the DRLs also express CD11c (Figure 10C), indicating that as with IDO1, HAAO is also expressed in DCs. Further supporting the role of HAAO expressed in the DRLs for neuropathic pain development, we found that intrathecal injection of a pharmacological inhibitor of HAAO (4-chloro-3-hydroxyanthranilate) (45) reduced mechanical allodynia (Figure 10E).

**Figure 11:**
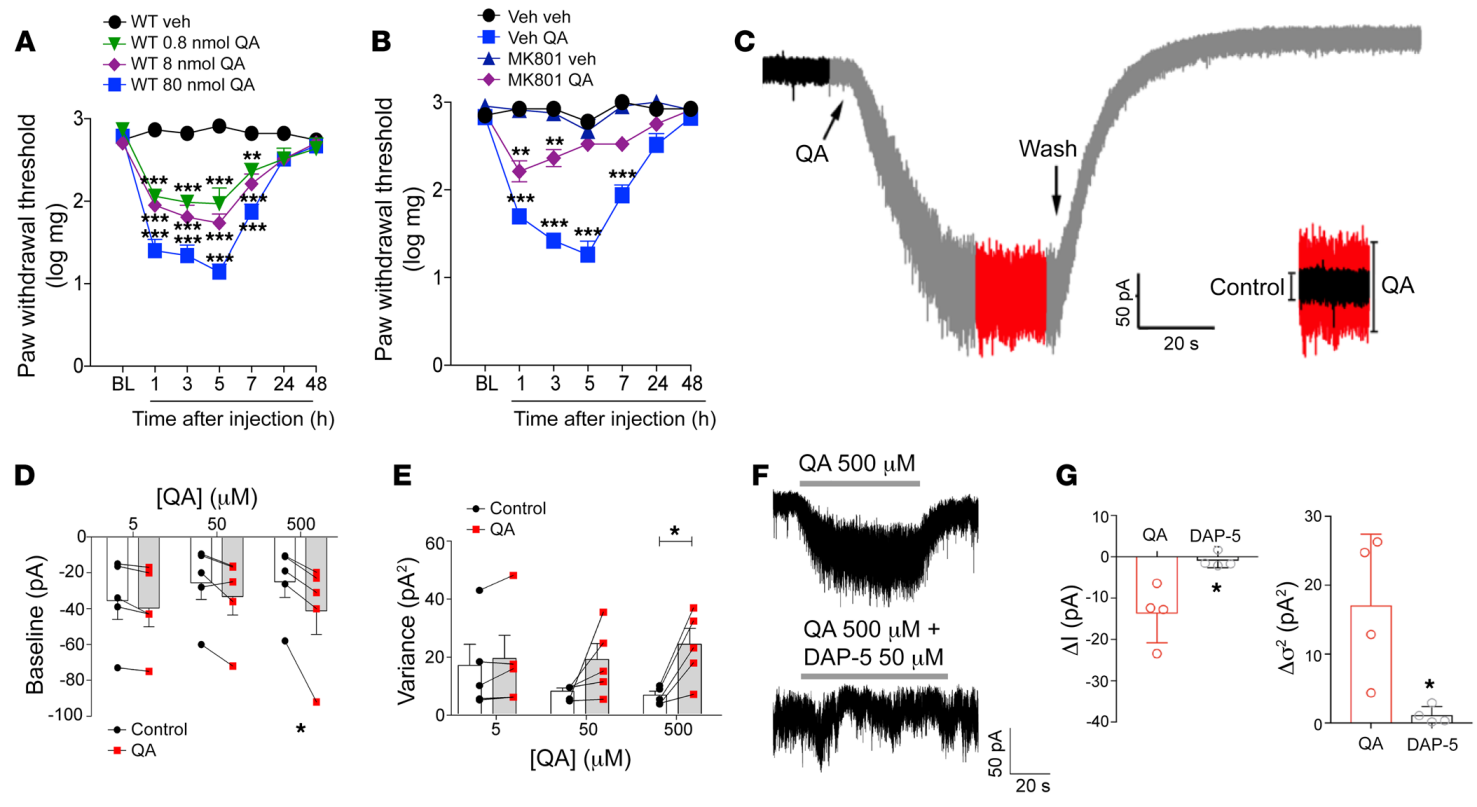
The pronociceptive activity of QA depends on upmodulation of NMDA currents. (**A**) Mechanical nociceptive thresholds were evaluated before and up to 48 hours after intrathecal (i.t.) injection of quinolinic acid (QA; 0.8–80 nmol) or vehicle (saline) in WT mice (*n* = 5). (**B**) Mechanical nociceptive threshold was evaluated before and up to 48 hours after i.t. injection of QA (80 nmol) in mice pretreated with MK801 (10 nmol) or vehicle (*n* = 4–5). (**C**) Representative trace and (**D**) quantification of QA (5–500 μM) eliciting an inward current (*n* = 5) and promoting an augmentation in peak-to-peak noise variance (**E**, *n* = 5) in spinal cord neurons. (**F** and **G**) Both effects were antagonized by the NMDA antagonist DAP-5 (50 μM, *n* = 4). Data are expressed as mean ± SEM. *P < 0.05, **P < 0.01, ***P < 0.001 versus vehicle, treatment, or current baseline by 2-way ANOVA with Bonferroni’s post hoc test (**A** and **B**), paired 2-tailed Student’s *t* test (**D** and **E**), or unpaired 2-tailed Student’s *t* test (**G**).

Since genetic and pharmacological inhibition of HAAO led to a reduction in neuropathic pain and the levels of QA increase in the spinal cord after SNI induction, we hypothesized that QA in the spinal cord plays a likely role in this process. In support of this hypothesis, intrathecal QA injection was found to promote mechanical allodynia in naive WT mice in a dose-dependent manner (Figure 11A). Intrathecal QA injection also promoted comparable levels of mechanical allodynia in WT, *Ido1^–/–^*, *Haao*^+/+^, and *Haao^–/–^* mice (Supplemental Figure 16, A and B), suggesting QA as the last step of KYNPATH that mediates neuropathic pain. Enhanced glutamatergic NMDA transmission in the spinal cord was reported to perpetuate neuropathic pain (46). Since QA is a well-known activator of NMDA receptors (16), we next analyzed whether this mechanism could be valid for the pronociceptive effect of QA in the spinal cord. Notably, pronociceptive activity of intrathecally injected QA was prevented by treatment with MK801, an NMDA receptor antagonist (Figure 11B). MK801 also alleviated SNI-induced mechanical allodynia (Supplemental Figure 17). Neuronal patch-clamp recording in spinal cord slices was used to further explore the effects of QA and NMDA signaling. In whole-cell voltage clamp recordings, QA application triggered an inward current when applied to a slice (Figure 11C). This effect was reversible and concentration dependent (Figure 11, D and E, and Supplemental Figure 18, A and B). Along with the inward current, the peak-to-peak noise of the baseline current also increased in the presence of QA, manifesting as augmented variance of the recorded trace (Figure 11, C and E). To test whether QA effects on spinal cord neurons depend on NMDA receptors to promote inward current, we added an NMDA receptor antagonist, D-AP5, along with the QA to the bath. D-AP5 abolished the QA-induced current completely and increased the peak-to-peak noise (Figure 11, F and G), indicating that QA causes an increase in membrane conductance through NMDA receptors. Collectively, these results indicate that HAAO-derived QA mediates neuropathic pain after SNI via activation of NMDA receptors.

## Discussion

Peripheral nerve injury–induced neuropathic pain development depends on complex multifactorial pathophysiological processes (1, 42, 47). It is thought that understanding of the cellular and molecular mechanisms involved in the transition from acute to chronic neuropathic pain after peripheral nerve injury would be the best way to discover novel targets for its control and/or prevention (48, 49).

In this context, in the present study we investigated the potential role of KYNPATH in the development of neuropathic pain following peripheral nerve injury. We found that pharmacological and/or genetic inhibition of crucial canonical enzymes of KYNPATH (IDO1 and KMO) led to a reduction in established neuropathic pain. We also found that after peripheral nerve injury, an accumulation of IDO-1–expressing DCs in the DRLs had occurred, a process that promotes an increase in the spinal cord levels of Kyn. Kyn is subsequently metabolized into a potent pronociceptive molecule, 3-Hk, by KMO-expressing astrocytes. Finally, we also found that HAAO-dependent QA, one of the last KYNPATH metabolites, also mediates neuropathic pain development. These data indicate an important mechanism of neuron–immune glia crosstalk in the pathophysiology of peripheral nerve injury–induced neuropathic pain.

The participation of KYNPATH in the development of pathological pain has been investigated previously (18–20, 27, 50). For instance, IDO1 genetic deficiency and pharmacological inhibition were found to alleviate arthritic pain (50). Furthermore, we have also shown that the participation of IDO1 in the genesis of viral infections incites pain hypersensitivity (27). Regarding neuropathic pain, the participation of KYNPATH, at least of IDO1, is controversial (18–20). Whereas intrathecal treatment with 1-MT reduced mechanical allodynia triggered by peripheral nerve injury in rats supports our findings, *Ido1^–/–^* mice developed mechanical allodynia that resembled that of WT mice (18, 19), a finding that was in contrast to our data. Although the explanation for this discrepancy is not immediately apparent, we can speculate about some possibilities for such a discrepancy, including animal colonies with different microbiota and different methods for quantifying mechanical allodynia (up and down method versus absolute threshold). Nevertheless, similar to our findings, none of these studies detected expression of IDO1 in the spinal cord after peripheral nerve injury (18, 20), but they found upregulation of IDO1 in the DRGs in rats (20) and systemic plasma increases in Kyn levels in SNI mice (18). Based on our findings, we can suggest that the increase in levels of IDO1 expression in rat DRGs after peripheral nerve injury might be due to the contamination of the DRGs with DRLs, which are difficult to completely separate from the DRGs. Notably, the increase in the expression of IDO1 in DRLs is associated with the phenotype observed in *Ido1^–/–^* mice and Kyn levels in the spinal cord. For instance, at early time points after SNI, in which IDO1 expression is not significantly upregulated, mechanical allodynia in *Ido1^–/–^* mice developed normally. In fact, early development of mechanical allodynia in this model is mainly dependent on the direct injury and activation of primary sensory neurons (51). SNI-induced neuropathic pain is mainly characterized by the development of mechanical and cold allodynia (52). Interestingly, KYNPATH seems to be involved only in SNI-induced mechanical allodynia but not in cold allodynia. This finding is in line with recent evidence suggesting that neuroimmune interactions are important for SNI-induced mechanical allodynia, whereas these interactions have no role in the development of cold allodynia (52).

In this study, we provide additional evidence concerning the mechanisms by which IDO1 is upregulated in the DRGs after peripheral nerve injury. Indeed, we found that IDO1 is expressed in DCs that accumulate in the DRL. In agreement with that finding, accumulation of peripheral leukocytes in the DRL after peripheral nerve injury has been demonstrated (32, 53, 54). For instance, CD4^+^ Th1 cells generated in the dLNs of the peripheral nerve injury accumulate in the DRL and drive glial and neuronal events in the spinal cord that in turn mediate neuropathic pain development (32). Thus, our study provides evidence that not only T cells can accumulate in the DRLs, but accumulation of DCs can also occur. Although our data did not provide evidence about the exact origin of these IDO1-expresssing DCs that accumulate in DRL, the finding that the population of these cells also increased in the dLNs and the spinal cord circulatory system would indicate they might be generated peripherally and infiltrate the DRL. Nevertheless, we could not disregard the notion that meninges-resident DCs (55) could also move toward the DRL after nerve injury. It is important to point out that DCs could be involved in the development of neuropathic pain via mechanisms independent of the expression of IDO1. For instance, they could be involved in self-antigen presentation and the generation of activated T cells with a Th1 phenotype that has been implicated in the development of neuropathic pain. Furthermore, they could be also involved in the production of pronociceptive molecules, such as cytokines and chemokines, a process that has been described recently in the context of postsurgical pain (56). Further studies are necessary to elucidate these points.

Another intriguing finding of our study was the role of KMO expressed by astrocytes in the maintenance of neuropathic pain. KMO expression has been identified mainly in neurons and microglial cells, at least in the brain (57–60). Furthermore, the stimulation of primary cultures of microglia with lipopolysaccharide (LPS) has been shown to lead to an increase in KMO expression (20). On the other hand, evidence that KMO is expressed in astrocytes of the spinal cord and of the forebrain has been published (60). In this study, we used a considerable number of different approaches to show that in the spinal cord, KMO is mainly expressed and upregulated in astrocytes after peripheral nerve injury. Furthermore, the specific knockdown of KMO in spinal cord astrocytes led to a reduction in mechanical allodynia that was triggered by peripheral nerve injury, indicating a role for KMO in the development of neuropathic pain. Supporting the importance of KMO in maintenance of neuropathic pain, we also presented evidence that its main downstream metabolite, 3-Hk, is a very potent pronociceptive molecule. Although we did not explore the possible mechanism(s) that triggers the increase in KMO expression in vivo, we showed that MCM and TNF in vitro could promote this process. Thus, in vivo, it is possible that microglia-derived mediators (including TNF) could be important for KMO upregulation.

The fact that Kyn and 3-Haa, in contrast to 3-HK, induced weak production of mechanical allodynia in naive animals when compared with 3-Hk might suggest 2 possible mechanisms: (a) the increase in the pronociceptive metabolites (such as 3-Hk) is not exclusively dependent on the availability of Kyn from DCs but might be also dependent on the upregulation of KMO in the spinal cord and (b) 3-Hk is one of the final directly acting pronociceptive mediators. It is important to emphasize that targeting KMO, for instance with pharmacological inhibitors, besides inhibiting the formation of the pronociceptive 3-Hk could also divert KYNPATH into the formation of an antinociceptive molecule, Kyna (61). In fact, Kyna is considered a neuroprotective endogenous antagonist of NMDA with analgesic properties (61, 62). Additionally, Kyna has also been recently identified as an endogenous agonist of G protein receptor–coupled 35 (GPR35), which could also be a possible alternative mechanism to control pathological pain (63–65). Another important point that emerged from our results is the possible mechanisms by which 3-Hk promotes pain hypersensitivity. Although the direct biding sites for 3-Hk are not known, several studies indicate its capability of triggering the formation of toxic free radicals, especially superoxide and hydrogen peroxide, which lead to mitochondrial damage that in turn leads to neuronal dysfunction (66–69). Importantly, these mechanisms are important for the development of neuropathic pain (70–76). Therefore, we suggest that the increase in KMO-dependent 3-Hk in the spinal cord after peripheral nerve injury could be a possible driver of oxidative stress in neuropathic pain development.

Our last set of results indicate that besides KMO-derived 3-Hk, HAAO-dependent QA is also involved in the maintenance of neuropathic pain. Furthermore, in agreement with the well-recognized biological function of QA (16), pronociceptive activity in the spinal cord was found to also be dependent on glutamatergic NMDA receptors. Of note, evidence that 3-Hk and QA synergistically induced neuronal changes and death, which would occur in neuropathic pain, has been presented previously (77, 78). One would expect that inhibition of HAAO might cause an increase in the levels of upstream pronociceptive metabolites (such as 3-Hk); however, a previous study demonstrated lack of a massive accumulation of 3-Hk in the brain of *Haao*-null mice in an LPS model of brain inflammation (79). This low accumulation could be explained by the multiple secondary metabolic pathways that could alleviate the buildup. Our study also provides additional information about identification of the cellular source of HAAO/QA. Similarly to what we found for the case of IDO1, HAAO was also identified in DCs that accumulate in the DRLs after peripheral nerve injury. Notably, the lack of HAAO expression in the sham-operated mice might explain why the intrathecal injection of 3-Haa did not produce mechanical allodynia in naive animals since the exogenous 3-Haa is not metabolized into pronociceptive QA. These results also indicate the critical role of meningeal DCs as an important cellular source of KYNPATH enzymes and metabolites that affect neuronal function. To our knowledge, this event is a previously unrecognized neuroimmune interaction and could be also involved in additional diseases of the CNS. Although in the present study we focused on possible pronociceptive actions of the KYNPATH metabolites in the spinal cord that mediate neuropathic pain, we cannot ignore the possibility that they could also reach the DRGs and affect the function of primary sensory neurons.

Neuropathic pain can be triggered by several different types of nerve injury or diseases of the somatosensory nervous system (1, 80, 81). It varies from chronic pain caused by physical trauma, as was mimicked by the SNI model to metabolic disorders, such as diabetes; infection, such as human immunodeficiency virus (HIV); and chemical induction, such as cancer chemotherapy (82). Although some common pathophysiological mechanisms are involved in the development of these neuropathic pain subtypes, they also have specific unique mechanisms (47, 83–85). Although we did not investigate the participation of KYNPATH in other models of neuropathic pain, evidence exists indicating an upregulation of KYNPATH in some diseases in which neuropathic pain is present, such as diabetes (82) and HIV (86). For instance, an increased concentration of 3-Hk in the CNS of HIV-1 patients was reported previously (87). In addition, KYNPATH enhancement is generally observed in different types of cancer, an important clinical condition in which pain has a neuropathic component (88–90). Therefore, further studies will be necessary to address whether KYNPATH is a common mechanism among different types of neuropathic pain.

**Figure 12:**
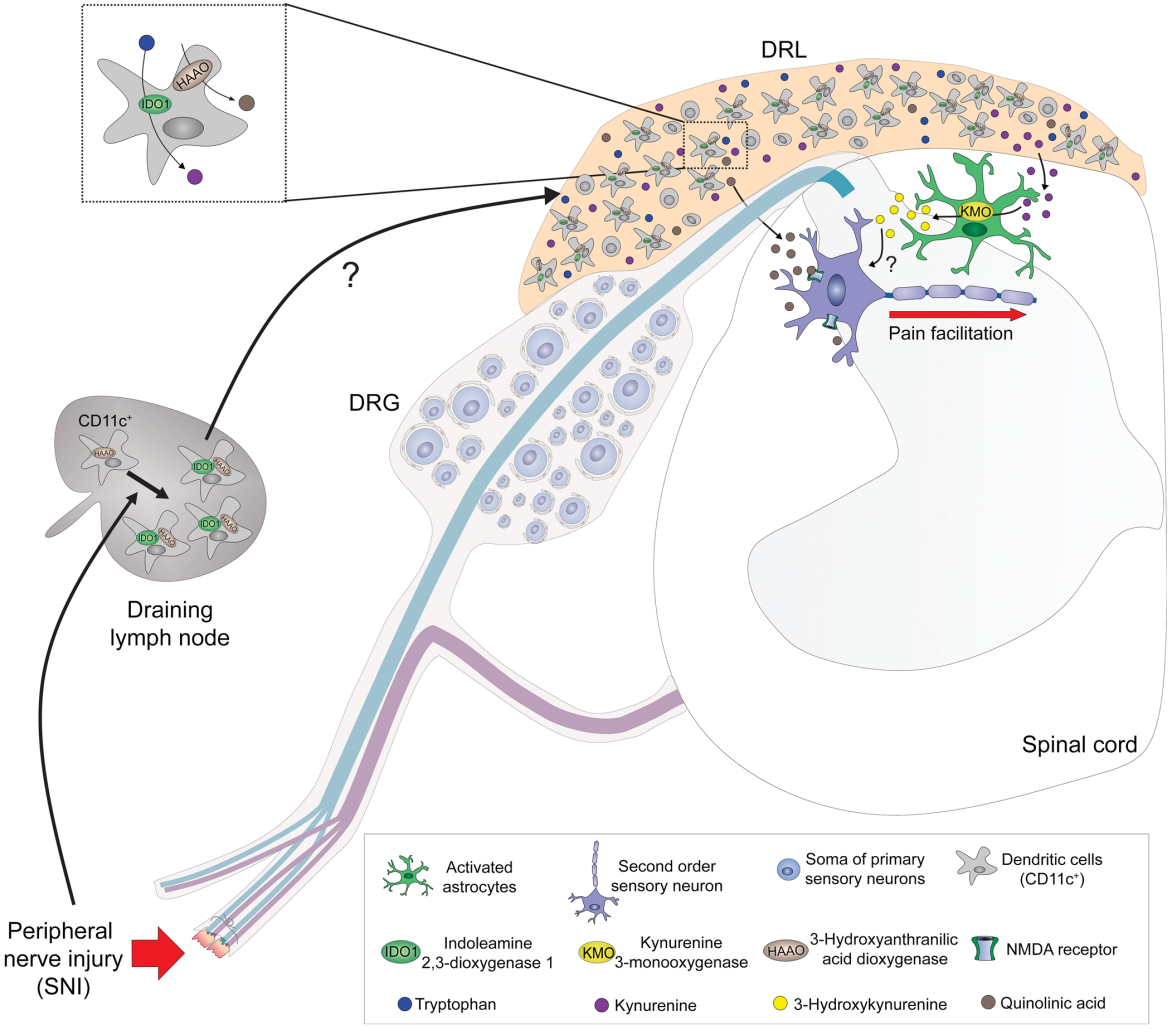
Schematic representation of the role of the kynurenine pathway in the maintenance of neuropathic pain. After peripheral nerve injury, there is an up regulation of IDO1 in dendritic cells (DCs), which accumulate in the dorsal root leptomeninges (DRLs), leading to an increase in the levels of kynurenine (Kyn) in the spinal cord. In the spinal cord, Kyn is metabolized by astrocyte-expressed KMO into a potent pronociceptive metabolite, 3-hydroxykynurenine (3-Hk). Additionally, HAAO-expressing DCs in (DRLs) might be responsible for the increase in the levels of QA in the spinal cord. QA is also a pronociceptive molecule in the spinal cord, acting through an enhancement of glutamatergic NMDA transmission and consequently to maintain pain hypersensitivity.

In summary, in the present study, we provided ample characterization of the role of the KYNPATH in the development of neuropathic pain. As depicted in Figure 12, after a peripheral nerve injury, IDO1 is upregulated in peripheral DCs, which might accumulate in the DRL, leading to an increase in the spinal cord levels of Kyn. Kyn is then metabolized locally by KMO-expressing astrocytes into the potent pronociceptive molecule 3-Hk. These data reveal what we believe is a previously unappreciated role for the KYNPATH as a critical link between the immune system (DCs), spinal cord glial cells (astrocytes), and neuronal sensitization to perpetuate neuropathic pain. These mechanisms may offer novel targets for drug development against this type of chronic pain.

## Methods

Full methods are available in the Supplemental Methods.

### Animals.

The experiments were performed in C57BL/6 male mice (WT, 20–25 g) and *Ido1^–/–^* (91) and *Haao^–/–^* mice (44). CD11c-DTR-eGFP (92), CD11c-eYFP (93), *CD11c*-*Cre*^+/–^ (The Jackson Laboratory, 8068) (93), and *Ido1^fl/fl^* transgenic mice were also used.

### Neuropathic pain model (SNI).

A model of persistent peripheral neuropathic pain was induced as previously described (24). The SNI consisted of an axotomy and ligation of the tibial and common peroneal nerves, leaving the remaining sural nerve intact. The common peroneal and the tibial nerves were tightly ligated with 5.0 silk and sectioned distal to the ligation, removing 2 ± 4 mm of the distal nerve stump.

### Behavioral nociceptive tests.

An investigator blinded to group allocation performed all the behavioral tests.

### von Frey filament test.

For testing mechanical nociceptive threshold, mice were placed on an elevated wire grid and the plantar surface of the ipsilateral hind paw stimulated perpendicularly with a series of von Frey filaments (Stoelting) with logarithmically increasing stiffness (0.008–2.0 g). The weakest filament able to elicit a response was taken to be the mechanical withdrawal threshold. The withdrawal frequency was calculated as the number of withdrawals after 10 applications of 0.16 g and 0.008 g von Frey filaments to the right hind paw (94).

### Statistics.

Data are reported as mean ± SEM. The normality of the distribution of data was analyzed by the D’Agostino-Pearson test. Two-way or 1-way analysis of variance (ANOVA) was used to compare the groups followed by Bonferroni’s *t* test (for 3 or more groups), comparing all groups, or when appropriate by unpaired, 2-tailed Student’s *t* test. Statistical analysis was performed with GraphPad Prism software. For electrophysiology data, the paired sample, 2-tailed *t* test was used. *P* values less than 0.05 were considered significant.

### Study approval.

Animal care and handling procedures were approved by the Committee for Ethics in Animal Research of the Ribeirao Preto Medical School USP (process nos. 045/2013 and 120/2018).

## Author contributions

AGM, GRS, and MD Fonseca designed experiments, performed experimental work, analyzed data, and prepared the manuscript. RMG, CEAS, and M Davoli-Ferreira performed experimental work related to flow cytometry and PCR and analyzed data. AD and RL performed experiments and analyzed the data related to electrophysiology. AHL, FIG, RLS, DAS, JT, GP, DSC, MBS, WAG, and RK performed experiments. ASM performed experiments related to RNAscope. LMM, GAB, and NPL performed experiments related to mass spectrometry and analyzed the data. LMA and NTC performed experiments related to lentivirus and shRNA. HL and LH performed experiments and provided important scientific comments. JCOC provided *Haao^–/–^* mice and important scientific comments. JCAF and FQC provided critical materials and comments. AM designed and supervised the study and provided critical materials and comments. TMC designed, directed, and supervised the study, interpreted data, and wrote the manuscript. All authors reviewed the manuscript and provided final approval for submission.

## Supplementary Material

Supplemental data

Supplemental video 1

Supplemental video 2

Supplemental video 3

## Version 1 10/13/2022

In-Press Preview

## Version 2 12/01/2022

Electronic publication
