# Supplementary material for: Meningeal dendritic cells drive neuropathic pain through elevation of the kynurenine metabolic pathway in mice
Source: J Clin Invest. 2022 Dec 1;132(23):e153805. doi: 10.1172/JCI153805 (PMC9711882; doi:10.1172/JCI153805)
Supplement: Supplemental data [file jci-132-153805-s143.pdf]

Supplementary Materials for

Meningeal dendritic cells drive neuropathic pain through elevation of the kynurenine metabolic pathway in mice

Alexandre G. M. Maganin†, Guilherme R. Souza†, Miriam D. Fonseca†, Alexandre H. Lopes Rafaela M. Guimarães, André Dagostin, Nerry T. Cecilio, Atlante S. Mendes, William A. Gonçalves[1]; Conceição E.A. Silva[1]; Francisco I. Gomes, Lucas M. Marques, Rangel L Silva, Leticia M. Arruda, Denis A. Santana, Henrique Lemos, Lei Huang; Marcela Davoli-Ferreira, Danielle S. Coelho; Morena B. Sant'anna, Ricardo Kusuda, Jhimmy Talbot, Gabriela Pacholczyk, Gabriela A. Buqui, Norberto P. Lopes, Jose C. Alves-Filho, Ricardo Leão, Jason C. O'Connor, Fernando Q. Cunha, Andrew Mellor and Thiago M. Cunha*.

*Correspondence to: thicunha@fmrp.usp.br

This PDF file includes:

Supplementary Methods

Supplementary references

Supplementary Figures 1 to 18

Supplementary Tables 1 and 2

Captions for Movies S1 to S3

Other Supplementary Materials for this manuscript include the following:

Movies S1 to S3

Supplementary Methods

Animals

The experiments were performed in C57BL/6 male mice (Wild Type, WT, 20-25 g) and mice deficient in the following protein: indoleamine 2,3 dioxygenase type-1 (*Ido1*$^{-/-}$)(1) and 3-hydroxyanthranilic acid dioxygenase (*Haao*$^{-/-}$)(2). We generated chimeric mice using bone marrow (BM) cells from CD11c-DTR-eGFP transgenic mice, which express diphtheria toxin receptor and eGFP under control of *Itgax* gene promoter (Itgax encoding CD11c)(3). We also used CD11c-eYFP mice that express enhanced yellow fluorescent protein (eYFP) under control of *Itgax* gene promoter(4). Mice lacking *Ido1* in CD11c^{+} cells (CD11c-Cre$^{+/-}$ *Ido1*$^{Flox/Flox}$) and control littermates (CD11c-Cre$^{-/-}$ *Ido1*$^{Flox/Flox}$) were generated by breeding CD11c-Cre$^{+/-}$ mice (Jax mice, 8068)(5) with *Ido1*$^{Flox/Flox}$ mice. *Ido1*$^{Flox/Flox}$ mice were generated from *Ido1*$^{tm1a(EUCOMM)Wtsi}$ mice purchased from European Mouse Mutant Archive-EMMA (EM:12287). *Ido1*$^{tm1a(EUCOMM)Wtsi}$ were bred with ACTB:FLPe mice (B6.Cg-Tg(ACTFLPe)9205Dym/J; JAX mice: 005703) to generate *Ido1*$^{Flox/Flox}$ conditional allele. Transgenic mice expressing the green fluorescent protein (GFP) in cells that express CX3C chemokine receptor 1 (CX3CR1$^{GFP/+}$)(6) were used for FACS sorting analyses of the microglia and astrocytes in the spinal cord. Local colonies of transgenic mice were then established and maintained on a C57BL/6 background at the animal care facility of Ribeirão Preto Medical School, University of São Paulo. Animals were taken to the testing room at least 1 h before experiments and were used only once. Food and water were available *ad libitum*. Four to twelve randomly assigned mice were used per experimental group per experiment. Mice were selected based on their genotype. All other criteria were not considered and as such, randomized. We were not blinded to mice genotypes or group allocations except where it is mentioned.

Neuropathic pain model (Spared nerve injury)

A model of persistent peripheral neuropathic pain was induced as previously described(7). Under isoflurane (2%) anesthesia the skin on the lateral surface of the thigh was incised and a section made directly through the biceps femoris muscle exposing the sciatic nerve and its three terminal branches: the sural, common peroneal and tibial nerves. The Spared Nerve Injury (SNI) comprised an axotomy and ligation of the tibial and common peroneal nerves leaving the remaining sural nerve intact. The common peroneal and the tibial nerves were tightly ligated with 5.0 silk and sectioned distal to the ligation, removing 2 ± 4 mm of the distal nerve stump. Muscle and skin were closed in two layers. For sham-operated mice, the sciatic nerve and its branches were exposed without lesioning. Then, mechanical pain hypersensitivity was evaluated up to 21 days after the surgery.

Inflammatory pain model

Mice received an injection of carrageenan (Cg- 100 µg/paw), CFA (10µL) or vehicle (saline) subcutaneously into the plantar region of the right hindpaw(8, 9). Then, mechanical(10) and thermal (heat)(11) pain hypersensitivity was determined at indicated time points after stimulus injection.

Behavioral nociceptive tests

An investigator blinded to group allocation performed all the behaviors tests.

Formalin nociception test

We assessed formalin-evoked nociception by injection of 20 μl of formalin (1%) into the dorsal surface of the right hind paw of mice. The time in seconds spent licking or flinching the injected paw was recorded and expressed as the total nociceptive behaviors in the early phase (0–10 min) and late phase (10–50 min) after the formalin injection(12).

Thermal Nociceptive Test.

The latency of paw withdrawal to radiant heat stimuli was measured using a Plantar Ugo Basile apparatus (Stoelting), as previously described(11). Mice can move freely in this apparatus on an elevated glass surface with plastic boxes above as the top cover. Mice were given a 1-h acclimation period before testing until they became calm and motionless. A calibrated infrared light source of high intensity was applied perpendicular on the plantar surface of each mouse's hind paw. The end point was characterized by the removal of the paw followed by clear flinching movements. Latency to paw withdrawal was automatically recorded. Each hind paw was tested alternately with an interval of 5 min for four trials.

von Frey filament test

For testing mechanical nociceptive threshold, mice were placed on an elevated wire grid and the plantar surface of the ipsilateral hind paw stimulated perpendicularly with a series of von Frey filaments (Stoelting, Chicago, IL, USA) with logarithmically increasing stiffness (0.008–2.0g). Each one of these filaments was applied for approximately 3-4s to induce a paw-withdrawal reflex. The weakest filament able to elicit a response was taken to be the mechanical withdrawal threshold. The log stiffness of the hairs is determined by log10 (milligrams) and ranged from 0.903 (8 mg or 0.008 g) to 3.0 (1000 mg or 1 g)(10, 13). The withdrawal frequency was calculated as the

number of withdrawals out after 10 application of 0.16 g and 0.008 g von Frey filaments in the right hind paw(14).

Hot-Plate Test

The noxious heat thresholds of the hind paws were also examined using the Hot-Plate test. Mice were placed in a 10-cm-wide glass cylinder on a hot plate (IITC Life Science) maintained at 48°C, 52°C or 56°C. Two control latencies at least 10 min apart were determined for each mouse. The latencies in seconds for paws licking or jumping for each animal were recorded. To minimize tissue damage, a maximum latency (cut-off) was set at 20 s(15).

Acetone test

Mice were placed in a clear plastic box with a wire mesh floor and allowed to habituate for 30 minutes prior to testing. Then, 50 µl fluid (acetone) was sprayed on the plantar surface of the hind right paw using a syringe of 1 ml (Tuberculin slip tip, BD, Franklin Lake, NJ, USA). Paw nociceptive responses, defined as flinching, licking or biting of the limb were measured within 1 minute after the application of acetone(16).

Drugs Administration

The i.p. administration: the drugs were injected into the ventral portion of the mouse close to the abdominal midline using a 1 mL syringe with a needle length of 2.5 cm and 0.7 cm gauge.

***Intrathecal injection**:* The technique used for intrathecal injection was done as described previously(17) with modifications. Under isoflurane (2%) anesthesia, mice were securely hold in one hand by the pelvic girdle and inserting a BD Ultra-Fine® (29G) insulin syringe (BD, Franklin

Lakes, NJ, USA) directly on subarachnoid space (close to L4–L5 segments) of the spinal cord. A sudden lateral movement of the tail indicated proper placement of the needle in the intrathecal space. For all administrations was used 5 µl of volume. Then, the syringe was held in the specific position for a few seconds and progressively removed to avoid any outflow of the substances

Enzymatic assay for IDO activity

IDO activity was assayed as described in a previous report(18). In brief, spinal cord tissues (L4-L6 level) were homogenized with a Polytron homogenizer (Kinematica, Lucerne, Switzerland) in 1.5 volumes of ice-cold 0.14 M KCl-20 mM potassium phosphate buffer (pH 7). The homogenate samples were centrifuged at 7000 X g and 4°C for 10 min. An aliquot of supernatant was taken for the measurement of IDO activity. The reaction mixture contained 50 µl enzyme preparation and 50 µl substrate solution. The composition of the substrate solution was 100 mM potassium phosphate buffer (pH 6.5), 50 µM methylene blue, 20 µg catalase, 50 mM ascorbate, and 0.4 mM L-TRP. Post-incubation of the reaction mixture at 37°C, samples were acidified with 3% perchloric acid and centrifuged at 7000 X g and 4°C for 10 min. The concentrations of the enzymatic products were measured by using HPLC. Enzyme activity was expressed as the product content per hour per gram of tissue protein.

Measurement of plasmatic kynurenine concentration

Plasma concentration of kynurenine (Kyn) was measured by high-performance liquid chromatography (HPLC) with a spectrophotometric detector (TOSOH UV-8000) or fluorescence spectrometric detector (HITACHI, Tokyo, Japan) as described previously[10]. Briefly, separation was obtained with a reverse-phase column (Brave ODS 3 µm 150 mm × 4.6 mm; Alltech, IL,

U.S.A.) and a mobile phase (flow rate 0.75 mL/min) composed of 0.1 M sodium acetate, 0.1 M acetic acid and 1% acetonitrile. The fluorescence excitation and emission wavelengths were set at 270 and 360 nm, respectively. UV signals were monitored at 355 nm for Kyn.

Measurement of tissue kynurenines concentration

Chemicals & reagents: L-Kynurenine (Kyn), 3-hydroxykynurenine (3-Hk), kynurenic acid (Kyna), 3-hydroxyanthranilic acid (3-Haa), ascorbic acid (AA), formic acid (FA) and trifluoroacetic acid (TFA) were obtained from Sigma Aldrich (Steinheim, Germany). Methanol (MeOH; LC–MS grade), acetonitrile (MeCN, LC–MS grade) were obtained from Merck KGaA (Darmstadt, Germany). The internal standard (IS) was: 6-Chloro-DL-tryptophan (TRP_Cl; Goldbio, St Louis, USA). All standards, solvents and reagents used were of highest purity (LC–MS grade where available). The water used was purified by means of a Milli-Q system (Merck Millipore, Germany).

Preparation of standard solutions: Stock solutions were prepared individually for each standard in a final concentration of 10 mM: 3-Haa, and Kyna were dissolved in H2O/MeOH/FA/AA (50/50/0.1/0.02). Kyn and 3-Hk were dissolved in MeOH with 0.1% FA and 0.02% AA. A final standard master mix (1 mM of each analyte) was performed by mixing individual standard stock solutions with acidified mobile phase (0.2% FA/0.05% TFA/1% MeCN in H2O). Internal standard (IS) individual stock solution (TRP_Cl) was prepared at the concentration of 84 nM in H2O/MeOH/FA/AA (50/50/0.1/0.02). All standard stocks were prepared on ice and stored at -80°C (19) .

Preparation of calibration curves: Calibration curves were obtained by spiking aliquots of 50 µl of matrices (dorsal root ganglia or spinal cord samples) with 10 µl of the standard master mix

solution and 10 µl of IS. The linearity of the proposed method was checked over the concentration range of 1–1700 nM to all compounds. Calibration curves were found to be linear (>0.990) over the selected range.

Sample preparation: Lumbar dorsal horn of the spinal cord was accurately weighted and transferred to a 2000 µl eppendorf. The samples were processed as follow: 50 µl of acidified mobile phase and 10 µl of IS were added. To each of those mixtures 150 µl of ice-cold MeOH was added and subsequently homogenized using a TissueLyser II (Qiagen). To support protein precipitation, samples were allowed to rest in -20°C for 30 min. After centrifugation (20000 g, 4°C, 15 min) supernatants were removed and evaporated to dryness under a gentle stream of nitrogen and dried extracts were reconstituted in 50 µl of acidified mobile phase. The quantification range was from 1 to 1700 nM.

LC–MS/MS system: The UPLC®-MS/MS analyses were performed using an Acquity UPLC (Waters, Milford, MA, USA) coupled to an Acquity TQD detector equipped with an ESI interface and a Kinetex F5 (50 × 2.1 mm, 1.7 µM) column. The mobile phase was composed of 0.2 % formic acid in water (v/v) (A) and 0.2 % formic acid in acetonitrile (v/v) (B) and it was pumped at a flow rate of 0.4 mL min-1. The column temperature was maintained at 40°C and the gradient elution program was performed as follows(19): 0.0 min (3% B), 0.3 min (3% B), 0.8 min (30% B), 1.8 min (60% B), 2.5 min (60% B), 3.0 min (95% B), 4.4 min (95% B), 4.5 min (3% B) and 7.5 min (3% B). The samples were conditioned at 10 ºC in the auto-sampler. The injection mode applied was the full-loop, using 10 µL of injection volume. Data were acquired by MassLynx v4.1 software and processed for quantification by QuanLynx V4.1 (Waters). The following generic source conditions were used in positive ionization mode: capillary voltage, 2.5 kV; cone, 10 V; desolvation temperature, 300°C; source temperature, 150°C, desolvation gas flow (N2), 750 L/h;

cone gas flow, 75 L/h; collision gas (argon), 0.16 mL/min. Multiple reaction monitoring (MRM) was used for the quantification of each compound, the specific metabolite transitions are presented in **Supplementary Table 1**. The MS conditions for each analyte were determined via direct infusion of individual standard.

Quinolinic acid quantification by UPLC-MS/MS

Lumbar dorsal horn of the spinal cord was accurately harvested, weighted deidentified and shipped to PsycoGenics Inc. for blinded analysis of QA. Spinal cord tissue samples was homogenized using a FastPrep 96 system (MP Biomedicals, LLC., Santa Ana, CA, USA) in combination with Covaris microTUBE containing AFA Fiber (Covaris Inc., Woburn, MA, USA). Spinal cord tissue preloaded and weighed in Covaris tubes with AFA fibers was diluted (3x based on masses exceeding 23 mg with a 0.2% acetic acid solution containing labelled internal standards) and homogenized for 20 minutes at refrigerated temperatures. Resultant solutions were then added into Vivaspin 2 filters from Sartorius (Sartorius, Stonehouse, Gloucestershire, UK) (2k MVCO) for ultrafiltration by centrifugation at 13,500 g for 60 minutes at 4°C. Resultant solutions were directly injected into a Waters (Milford, MA, USA) Acquity UPLC system equipped with an YMC™ ODS-AQ™ 2 mm × 100 mm, 3 µm particle column. QA analyte was detected by a Waters Quattro Premier XE triple quadrupole mass spectrometer (Waters, Milford, MA, USA), operating in the MS/MS mode. Column and pre-column tubing are maintained at 40 °C while eluting kynurenine metabolites with a mobile phase consisting of an aqueous component (A: 0.5% formic acid in milliQ water) and an organic component (B: 1% formic acid in acetonitrile). Gradient elution included a 2 min hold at 100% A followed by a shallow gradient of 0%–30% B over 4.4 min. Later eluting materials are then brought off the column using a stronger gradient of 30%–50% B over

0.5 min with a total run time of 9 min. Quantification of the analyte was determined by correction vs. stable labelled internal standards of known concentrations and averaged on triplicate determinations where possible based on sample filtrate volume. Limits of quantification are determined based on a signal to noise ratio of 10:1 and found to be below required limits for analysis of this analyte in rodent samples.

Standard curve was prepared using pure quinolinic acid (QA) dissolved in 0.2% acetic acid to correct for sample and instrument variability. In addition corresponding stable label internal standard (either deuterium or carbon 13 analogs of the indicated analyte) at a known constant concentration was incorporated into the samples and standards to correct for sample and instrument variability and analyte recovery from sample preparation. The concentrations of QA obtained in the samples were normalized by sample weight.

Reagents

The following drugs were used in this study: 1-Methyl-tryptophan (#860646), norharmane (#N6252), Ro-618048 (#SML0233), L-kynurenine (Kyn) (#K8625), 3-Hydroxy-DL-Kynurenine (3-Hk) (#H1771), 3-hydroxyanthranilic acid (3-Haa) (#H9391), quinolinic acid (QA) (#P63204) and Complete Freund's Adjuvant – CFA; MK801 (Tocris Bioscience, Bristol, United Kingdom), diphtheria toxin (Dtx, #322326, Merck-millipore-Calbiochem San Diego, CA, USA), Kappa (k)-Carrageenan) (BDH Biochemicals, Poole, England). The 4-chloro-3-hydroxyanthranilate (#EN300-22755, Enamine Ltd., Ukraine).

Western blot analysis

Mice were terminally anesthetized at indicated times after SNI or sham surgery and ipsilateral dorsal horn of the spinal cord tissues, draining lymph nodes (inguinal and popliteal) or dorsal root ganglion (DRGs) with dorsal root leptomeninges (DRL) ipsilateral to the lesion were harvested. Part of the experiments was performed in PBS perfused mice. The samples were homogenized, and the expression of IDO1 and KMO were evaluated using western blotting analyses. Primary culture astrocytes or cultured U87 cells were also used for western blotting analyses. Briefly, samples were homogenized in a lysis buffer containing a mixture of protease inhibitor (Protease Inhibitor Cocktail Tablets - Roche Diagnostics). Proteins were separated using SDS-polyacrylamide gel electrophoresis (SDS-PAGE-4-12%) and transblotted onto nitrocellulose membranes (Bio-rad, California, USA). The membranes were blocked with 5% dry milk (overnight) and incubated overnight at 4°C with a mouse monoclonal antibody against IDO1 (# SC-365086 - E7 1.500, Santa Cruz Biotechnology, Dallas, Texas, USA), KMO (# NBP1-44263 1:1000, Novus Biologicals, Littleton, Colorado, USA), and a mouse monoclonal antibody against human KMO (# 60029-1-Ig 1:1000, Thermo Fisher Scientific, Massachusetts, EUA). The membranes were washed and incubated for 1 h at room temperature with an HRP-conjugated secondary antibody (1:10000; Jackson ImmunoResearch, PA, USA). Immunodetection was performed using an enhanced chemiluminescence light-detecting kit (Amersham Pharmacia, Biotech, Little Chalfont, UK) for 2 min. A mouse monoclonal antibody against β-actin (# A5316 1:10000; Sigma Aldrich, Saint Louis, Missouri, USA) and a rabbit monoclonal antibody against GAPDH (# 97166 1:5000, Cell Signaling Technology, Massachusetts, EUA) were used for loading controls. Images were used as representative blots. The capture of images was performed with Quemi TM-Doc XRS apparatus. Densitometric data were measured following normalization to

the control (house-keeping gene) using Scientific Imaging Systems (Image labTM 3.0 software, Biorad Laboratories, Hercules CA).

U87MG cells culture and differentiation

U87MG cells (ATCC® HTB-14™), a human malignant glioblastoma (astrocytoma), was placed 1 x 10^5/wells in 24 wells plate with Dulbecco's Modification of Eagle's Medium – DMEM (Corning Incorporated, Nova York, EUA) and 10% inactivated fetal bovine serum plus 1% Penicillin/Streptomycin. After placed, the cells were treated with 1 M of all-trans retinoic acid-ATRA (Sigma Chemical, St. Louis, MO, USA) for 7 days for astrocyte differentiation as previous described(20). At the end, FACS analyses revealed that 98% of cultured cells express GFAP. After differentiation, cells were stimulated with human TNF recombinant (10 ng/ml) (Sigma-Aldrich Corporation, Missouri, EUA) for 24 hours.

Construction of pLenti-GFAP-shKMO

The lentiviral vector used for cloning of shRNA targeting kynurenine 3-monooxygenase (KMO) was derived from the pLenti-GFAP-shAct1 plasmid, kindly provided by Dr Guang-Xian Zhang(21). For pLenti-GFAP-shKMO construction, the shACT1 was replaced by the 21nt sequence GCACTGAATGCCTGCTTTCTT shRNA targeting murine KMO (NM_133809.1), designed using siRNA Wizard v3.1™ (Invivogen, San Diego, USA). The entire region of siRNA was synthesized as oligonucleotides, annealed and inserted in the previously XhoI/EcoRI digested plasmid, under standard procedures. The constructed plasmid sequence was confirmed by DNA

Sanger sequencing. The vector without insertion of shKMO was used as a negative control. Oligos used for shKMO construction are listed below.

Forward: TCGAGAAGGTATATTGCTGTTGACAGTGAGCGAGCACTGAATGCCTGC TTTCTTTAGTGAAGCCACAGATGTAAGAAAGCAGGCATTCAGTGCTGCCTACTGCCT CGG

Reverse: AATTCCGAGGCAGTAGGCAGCACTGAATGCCTGCTTTCTTTACATCTG TGGCTTCACTAAAGAAAGCAGGCATTCAGTGCTCGCTCACTGTCAACAGCAATATAC CTTC

Virus packaging, concentrating and tittering

The pLenti-shKMO (or empty vector) and two helper plasmids pPAX2 (Addgene #12259) and pMD2 (Addgene #12260), were used to transfect into HEK293FT cells using Lipofectamine 3000 (Thermofisher). After 6 hours of incubation, the medium with plasmids was replaced by fresh Dulbecco's modified Eagle's medium supplemented with 10% fetal bovine serum, 2mmol/l L-Glutamine, 100 IU/ml penicillin, and 100μg/ml Streptomycin. Supernatants were harvested after 30 hours and filtered through a 0.45μm membrane filter. The filtered supernatant was submitted to ultracentrifugation (29000 RPM, 2h, 4C). The pellet containing lentivirus was resuspended in PBS/BSA1%, and aliquots were stored at −80°C. Viral titers were assayed by Lenti-X qRT-PCR (Clontech) and titers were adjusted to 1×108 copies/ml before injection.

Intraspinal injection of Lentivirus vector

Intraspinal injection of Lentivirus vector were performed as previous described with some modifications(22). The mice were deeply anaesthetized by intraperitoneal (i.p.) injection of

ketamine (100 mg kg^{-1}) and xylazine (10 mg kg^{-1}), after the mice was immobilized and attached in the rostral and caudal sites of the vertebral colum, and the skin was incised at Th12–L3. Paraspinal muscles around the left side of the interspace between Th13 and L1 vertebrae were spread and removed, the dura mater and the arachnoid membrane were carefully incised using the tip of a 30G needle to make a small cavity to allow the microcapillary Femtotip (Eppendorf, NY, USA) insert directly into the dorsal region of spinal cord. The microcapillary was inserted with $1x10^7$ lentivirus solution through the small cavity. After microinjection, the skin was sutured with 4-0 silk, and mice were kept on a heating pad until recovery.

Intrathecal ShRNA against KMO

The short hairpin RNA targeting the murine expression of KMO (NM_133809.1) was designed and acquired from Genecopoeia (plasmid reference MSH037508-CU6). The *in vivo* transfection was performed using InVivo JetPei reagent (Polyplus) and administered intrathecally as described previously(23) starting at 10 up to 13 days after SNI induction.

Real-time RT-PCR

After collection of tissue from the region comprised between lumbar segments (L3-L6) of the dorsal horn of spinal cord, draining lymph nodes ipsilateral to the lesion, primary culture astrocytes or cultured U87 cells were rapidly homogenized in Trizol (Sigma) reagent at 4°C. Then, total cellular RNA was purified from tissue, according to the manufacturer's instruction. RNA concentration was determined by optical density at a wavelength of 260 nm by means of the apparatus NanoVue ® (*GE Helthcare*). One microgram of total RNA was transcribed to cDNA by reverse transcriptase enzyme action Improm Pre-II ® (Promega, Madison, Wisconsin, USA).

Quantitative RT-PCR reaction in real time was done on an ABI Prism ® 7500 Sequence Detection System (*Applied Biosystems*), using System SYBR-green fluorescence (*Applied Biosystems, Warrington, UK*) for the quantification of amplification. RT-PCR was performed with the final volume of the reaction 6.25 µL and kept on 95 °C (10 min) and 40 cycles of 94 °C (1 min), 56 °C (1 min) and 72 °C (2 min). The melting curve was analyzed (65-95 °C) to verify that only one product was amplified. Samples with more than one peak were excluded. The results were analyzed by the method of quantitative relative expression $2^{-\Delta\Delta Ct}$ as previously described(24). The primer pair for mouse can be found at **Supplementary Table 2.**

Immunofluorescence

After appropriate times, animals were deeply anesthetized with ketamine and xylazine and perfused through the ascending aorta with PBS, followed by 4% paraformaldehyde. Spinal cord sections (60 µm, free-floating), DRGs plus DRLs (20 µm) or draining lymph nodes were washed in PBS (0.01 M, pH 7.4) 3 x 5 minutes and incubated in 1% BSA, dissolved in phosphate buffered saline with Triton X100 (PBST) at 0.1% for 1 hour. In some experiments related to DRLs, whole lumbar vertebrae containing spinal cord and DRGs (L3-L5) referring to sciatic nerve were obtained from SNI (14 days) and sham animals were fixed with 4% paraformaldehyde buffered with 0.1M phosphate solution, pH 7.2-7.4, for 4 hours at 4°C. Next, tissues were decalcified with 5% EDTA in 1X Dulbecco's PBS solution for 10 days at 4ºC and serially cryoprotected in 10%, 20%, and 30% sucrose serie diluted in 1X Dulbecco's PBS solution overnight each at 4°C. Subsequently, the sections were washed in PBS (0.01 M, pH 7 4) 3 x 5 minutes and then processed according to the technique of immunofluorescence labeling with overnight incubation at 4 °C with the polyclonal anti-Iba1 1:250 (# NB100-1028, Novus Biologicals, Littleton, Colorado, USA),

monoclonal anti-GFAP 1:500 (# MAB3402X, Millipore, Darmstadt, Germany), anti-NeuN 1:200 (# MAB377X Millipore, Darmstadt, Germany), anti-KMO 1:200 (# NBP1-44263, Novus Biologicals, Littleton, Colorado, USA), a mouse monoclonal antibody against IDO1 (# sc365086, clone E7, Santa Cruz Biotechnology, Texas, EUA), monoclonal antibody against CD11c 1:300 (#117308, Biolegend, California, EUA) and polyclonal anti-GFP conjugated Alexa 488 (# A211311, Life Technologies, California, EUA) and CD11c 1:300 (#117308, Biolegend, California, EUA). After incubation with the primary antibodies, the sections were washed in PBST 3 x 5 min and incubated at room temperature for 1 hour with Alexa fluor 488®goat anti-mouse or 594®donkey anti-rabbit IgG (Molecular Probes, Eugene, Óregon, USA). The sections were washed with PBS-T as described earlier, and mounted on glass slides, covered with cover slips with FluromountTM Aqueous Mounting Medium (Sigma, St. Louis, Missouri, USA). For spinal cord sections, images were acquired in SP5 confocal microscopy (Leica, Wetzlar, Germany) at 10, 20 and 40x magnification, and they represent a maximum intensity projection of a z-stack scanning. Image size was 1024 x 1024. For DRGs/DRLs sections, images were acquired in Zeiss LSM 780 confocal AxioObserver (Carl Zeiss, Germany) with an objective lens 40x (N.A 1.3 oil immersion), appropriate pairs of filters and dichromatic mirror. The excitation/emission wavelength light source, provided by a fiber-coupled diode with λ = 488 – 25mW/540, 594 – 2mW/667 and 405 - 30mW/ 453 nm without overlap. A 8-bit images were collected by XY-scanning stage with 4 Tile Scan (independent mosaic scan acquisition channel) and the z-direction with 13 slices (24 µm step size) scaling 0.35 µm x 0.35 µm x 2.0 µm and image size was 672.52 x 672.52. The image-analysis software platform Fiji/Image J (Fiji Is Just ImageJ) were applied to image dimension adjustment, small correction of brightness and cell count. Where mentioned, the number of cells expressing IDO1 relative to the number of cells (DAPI+) was performed in the DRLs.

RNAscope® in situ hybridization combined with Immunofluorescence

Whole lumbar vertebrae containing spinal cord and DRGs (L3-L5) referring to sciatic nerve were obtained from SNI (14 days) and sham animals were fixed with 4% paraformaldehyde buffered with 0.1M phosphate solution, pH 7.2-7.4, for 4 hours at 4°C. Next, tissues were decalcified with 5% EDTA in 1X Dulbecco's PBS solution for 10 days at 4ºC and serially cryoprotected in 10%, 20%, and 30% sucrose serie diluted in 1X Dulbecco's PBS solution overnight each at 4°C. Sections (20 μm thickness) were washed two times in 1X PBS, serially dehydrated in 50%, 70%, and 100% ethanol for 5 min each and stored in 100% alcohol until assay run. RNAscope® *in situ* hybridization assay for fresh-frozen samples was performed according to manufacturer's instructions of RNAscope® Multiplex Fluorescent Reagent kit v2 (Cat. No. 323100, Advance Cell Diagnostics (ACD), Hayward, CA, USA). All incubation steps were performed on the HybEzTM hybridization system (ACD). Briefly, in the pretreatment steps, samples were incubated with hydrogen peroxide reagent during 10 min at RT and washed with ultra-pure water twice. Then, samples were incubated with protease IV solution for 30 min at RT and washed with 1X PBS twice. Custom mouse Haao RNAscope probe were designed and purchased from ACD. HAAO probe targets the region 332-1252 (Accession number: NM_025325.2) of the HAAO sequence with 20 pairs of ZZ-target probes. The probe was applied and let hybridized for 2 h at 40°C. The amplification and detection steps were performed according to the manufacturer's advice. Detection was developed using Opal® Dye (Akoya Biosciences) fluorophores diluted in TSA buffer (1:750, Cat. No. 322809, ACD) for 30 min at 40°C. After hybridization steps, sections were washed with TBST Wash Buffer under gentle agitation and blocked with 10% normal serum in TBS-1% BSA for 30 min at RT. Primary antibodies against CD11c 1:300 (#117308, Biolegend, California, EUA) and HAAO 1:400 (#bs-11695R-A488, Bioss Inc, Massachusetts, EUA) diluted

in TBS-1%. Kidney sections were used as a positive control of the *Haao* probe. BSA were incubated overnight at 4ºC. Sections were washed five times with T-BST Wash Buffer and secondary antibodies were performed. In the end, the sections were incubated with DAPI solution (ACD) and images were acquired in Zeiss LSM 780 confocal (Carl Zeiss, Germany) as described above. The number of cells expressing *Haao* relative to the number of cells ($DAPI^+$) was performed in the DRLs.

Generation of bone marrow-chimeric mice

Recipient mice were exposed to 9-gray total-body irradiation using a X-ray source (Mark I, model 25). One day later, the animals were injected via tail vein with 4×10^6 bone marrow (BM) cells freshly collected from donor mice. The cells were aseptically harvested by flushing femurs with Dulbecco's PBS (DPBS) containing 2% fetal bovine serum. The samples were combined, filtered through a 40 µm nylon mesh, centrifuged, and passed through a 25-gauge needle. Recovered cells were resuspended in DPBS at a concentration of 5 × 106 vial nucleated cells per 200 µl. Irradiated mice transplanted with this suspension were housed in autoclaved cages and treated with antibiotics (10 mg of ciprofloxacin per milliliter of drinking water given for 2 weeks after irradiation)(23). Mice were subjected to SNI surgery 2 months after transplantation. The chimeric mice were generated as follow (donor → recipient): (1) WT and $Ido1^{-/-}$ recipient mice transplanted with BM from WT mice (WT→$Ido1^{-/-}$ and WT→WT), (2) $Ido1^{-/-}$ recipient mice transplanted with $Ido1^{-/-}$ donor mice ($Ido1^{-/-}$ →$Ido1^{-/-}$). At 60 days after transplantation, SNI was induced in chimeric mice.

Generation of $CD11c^{DTR/hema}$ chimeric mice and DCs depletion

For *in vivo* depletion of DCs we performed a previously described method(25, 26), with modifications. We used a chimera-based approach to avoid side effects during DCs depletion protocol. The experimental design for the generation of chimeric mice is shown in Fig. 2D. Briefly, WT mice were irradiated with a dose of 9.0 Gy. After 24 h, a total of 5.0 x 10^6 bone marrow-derived cells from CD11c-DTR mice were injected intravenously in the irradiated mice. After 8 weeks, the reconstituted chimeric mice (CD11c$^{DTR/hema}$ mice) were randomly separated into for groups and submitted to following experimental protocols. For conditional DC ablation [CD11c$^{DTR/hema}$ mice chimeras were inoculated i.p. with 16 ng DTx/g body weight every second day from day 0 up to day 12 after SNI induction.

Dendritic cells sorting from draining lymph nodes

At 14 days after SNI, draining lymph nodes (DLNs; inguinal and popliteal) from CD11c-eYFP mice were pooled (5 mice per sample) homogenized in RPMI 1640 media pH 7.4 (GIBCO), then digested with 1 mg/ml of collagenase A for 30 min at 37°C. Single cell suspensions were prepared using a 70 µm-Cell strainer (Corning– Durhan, USA) nylon mesh, layered over RPMI 1640 containing 10% FCS and centrifuged at 450 *g* for 10 min at 4°C and the supernatant was discarded. To purify total CD11c$^+$ DCs, single cell suspensions from lymph nodes were further sorted (eYFP$^+$ cells) in a FACSAria III sorter. Over 5% of the sorted cells obtained were CD11c$^+$. Sorted cells were submitted to RNA extraction, reverse-transcribed with High Capacity Kit (Life Techonologies) and analyzed by quantitative RT-PCR with a Step One Real-time PCR system as described above (Applied Biosystems).

Microglia and astrocytes cells sorting from spinal cord

Astrocytes and microglia were isolated from the spinal cord as previously described(27–29); with minor modifications. Briefly, the spinal cord between lumbar segments (L4-L6, pooled of 8 animals) were collected from CX3CR1$^{GFP/+}$ mice and incubated isolated in 1 ml of RPMI medium containing 1mg/ml of collagenase D (Roche, cat. no. 1-088-874) for 30 minutes at 37ºC. After this time, the tissues were passed through a cell strainer (100 µm), followed by centrifugation with RPMI medium containing 10% fetal bovine serum (FBS, Gibco). Cells were obtained and resuspended in 10 ml of 33% Percoll (GE Health Care) solution. After centrifugation, cells were resuspended and incubated in PBS 0.1M (2% FBS) containing cell viability dye – APCH7 (cat. no. 65-0865-14, eBioscience, 1:5000) and anti-ACSA2 - PE (IH3-18A3, Miltenyi Biotec, 1:250), a specific marker of astrocytes, for 15 minutes at 4ºC. Finally, cells were isolated using FACSAria III (BD Bioscience) and then the pellets obtained were resuspended in lysis buffer to isolate mRNA. The data were analyzed using FlowJo 10 software (Treestar, Ashland, USA). The gating strategy used to perform cell sorting from spinal cord is depicted in **Fig. 8**. Expression of specific cell markers, *Cx3cr1* for microglia and *Gfap* for astrocytes and *Kmo* were analyzed by real-time PCR (see specific method) in both populations.

Flow cytometry acquisition.

DLNs (inguinal and popliteal) were removed from CD11c$^{DTR/hema}$ mice treated with Dtx or vehicle after 14 days of SNI or sham surgery, teased into single-cell suspensions, and filtered through a 70-µm cell strainer (Corning # 431751 – Durhan, USA) and proceed as described above. DCs (CD11c-eGFP^{+}) cells were detected and the frequency of cell group was determined in BD FACSVerse (BD Biosciences), and data were analyzed using FCS Express (De Novo Software™).

Generation of bone marrow-derived dendritic cells and transfer

Bone marrow-derived dendritic cells (BMDCs) were generated from the differentiation of bone marrow cells. Briefly, animals were killed and the femur bone carefully dissected. In sterile environment, the bone epiphyses were cut and a 20g needle, coupled to a complete RPMI-1640 filled syringe (supplemented with 10% fetal bovine serum, glutamine (2 mM), streptomycin-0.01 mg/mL, penicillin-10 U / mL, antifungal-amphotericin B- 30 μg / mL) was inserted at one end. Next, the internal cavity of the bone was washed away, removing the entire bone marrow. Cells were added to the culture plates at the density of 2.0 x 10^6 cells / dish in a final volume of 10 mL of complete RPMI-1640 supplemented with 20 ng/mL GM-CSF (granulocyte-macrophage colony stimulating factor; R&D Systems). After 3 days, another 10 mL of medium supplemented with the same concentration of GM-CSF was added. At the end of 7 days, the cells were collected from the plate and used for the remaining experiments(26). BMDCs (5.0 x 10^6; i.v.) were transferred to WT or *Ido1*$^{-/-}$ mice one day after SNI surgery.

Primary Astrocytes Culture and Microglia Conditional Medium

For the primary culture, the astrocytes were obtained by the microbeads isolation on the cortex of newborn mice (1 to 3 days). The newborn mice were anesthesia in ice, and the cortex was collected in Dulbecco's Modification of Eagle's Medium – DMEM (Corning Incorporated, Nova York, EUA) with penicillin and without bovine fetal serum. After isolation, the cells of cortex were dissociated and marker with anti-GLAST (ACSA-1; cat n. 130-095-826) biotinilad antibody, after with anti-biotin microbeads (cat n. 130-095-826; Miltenyi Biotec, Bergisch Gladbach, Germany). The cells were passed in MS Columns (cat n. 130-042-201; Miltenyi Biotec, Bergisch Gladbach,

Germany), and only the ACSA-1 positives cells stay attached in the columns. The isolated astrocytes were placed 5x10^4 cells/well in a 24 well place for 7 days with DMEM (Corning Incorporated, Nova York, EUA) (high glucose, 10% heat-inactivated fetal bovine serum and 1% Penicillin/Streptomycin). After 7 days, the astrocytes were stimulated with recombinant mouse TNF (cat n. T7539; 10 ng /ml) (Sigma-Aldrich Corporation, Missouri, EUA) or Microglia Conditional Medium MCM (300 µl) for 24 hours. To determinate the purity of culture, the cells were marker with anti-GFAP-PE (561483, California, EUA, BD Biosciences) for flow cytometry acquisition. For the MCM, microglia were obtained by the same protocol of astrocytes primary culture. However, the cells of newborn mice cortex were dissociated and marker with anti-CD11b-PE antibody (cat n. 553311; BD Biosciences, California, EUA), after with anti-PE microbeads (cat n. 130-048-801; Miltenyi Biotec, Bergisch Gladbach, Germany). The cells were passed in MS Columns (cat n. 130-042-201; Miltenyi Biotec, Bergisch Gladbach, Germany), and only the CD11c positives cells stay attached in the columns. The isolated microglia were placed 10^5 cells/well in a 24 wells place for 5 days with DMEM (Corning Incorporated, Nova York, EUA) (high glucose + 10% heat-inactivated fetal bovine serum + 1% Penicillin/Streptomycin). After 5 days, the microglia were stimulated with LPS (cat n. L2630; 100 ng /ml) (Sigma-Aldrich Corporation, Missouri, EUA) for 30 min, washed and stayed in medium for 24 hours to obtain the MCM.

Electrophysiology

Spinal cord slices were obtained from the lumbar (L4-6) of male and female naive mice. The animals were anesthetized using isoflurane, quickly decapitated and the spinal cords removed and

placed in ice-cold cutting solution (in mM: 92 NMDG-Cl, 2.5 KCl, 1.25 NaH_2PO_4, 30 $NaHCO_3$, 20 HEPES, 25 glucose, 2 thiourea, 5 Na-ascorbate, 3 Na-pyruvate, 0.5 $CaCl_2$, 10 $MgSO_4$. pH to 7.3–7.4 with HCl and the meninges removed to dissect the lumbar spinal cord(30). The spinal cords were cut in 500 μm slices using a vibratome VT-1000, left for 12 -15 minutes at 34ºC in cutting solution, transferred to a mixture of 50% cutting solution and 50% recording solution (see composition below) at 34°C for 40 minutes and maintained at room temperature in the same solution until recording.

Substantia gelatinosa was visualized under oblique illumination through a 60 x water-immersion objective (Olympus). Slices were constantly perfused with a Mg-free recording solution (in mM: 127 NaCl, 3.5 KCl, 1.2 KH_2PO4, 26 $NaHCO_3$, 10 glucose, 2.4 $CaCl_2$. pH 7.3 - 7.4 when bubbled with 95%O_2/5%CO_2 mix) at room temperature with picrotoxin (5 μM) and strychnine (1 μM). Recordings were made through borosilicate microelectrodes fabricated in a Sutter P-87 horizontal puller with resistance ranging from 4 - 7 MΩ when filled with potassium gluconate internal solution (138 K Gluconate, 0.5 EGTA, 10 HEPES, 8 KCl, 4 ATP-Mg, 0.3 GTP-Na, 10 Phosphocreatine, pH 7.4). Neurons were identified by the presence of action potential firing. Signals were acquired in voltage clamp mode at a holding potential of -70mV, with a 10 kHz sampling rate and low-passed filtered at 2.9 kHz (Bessel) using a HEKA EPC10 amplifier.

QA was diluted in the recording solution to make a stock concentration of 10 mM, made fresh every day, and then diluted in recording solution to final concentrations of 5, 50 or 500 μM. Drug perfusion was gravity fed (1 ml/min) and different concentrations were selected using an 8-way Valvelink controller (Automate Scientific) and applied directly on the cell using a custom-made 6-way manifold with a 250 μm ID MicroFil (World Precision Instruments) needle as output. Quinolinic acid was applied during a period of 2 minutes and its washout lasted, at least, 5 minutes.

Data analyses and statistics

Data are reported as the means ± s.e.m. The normal distribution of data was analyzed by D'Agostino and Pearson test. Two-way analysis of variance (ANOVA) was used to compare the groups and doses at the different times (curves) when the responses (nociception) were measured after surgery or treatments. The analysed factors were the treatments, the time, and the time versus treatment interaction. If there was a significant time versus treatment interaction, One-way ANOVA followed by Bonferroni's t-test was performed for each time. Alternatively, if the responses (eg. protein expression, mRNA expression) were measured only once after the stimulus injection, the differences between responses were evaluated by one-way ANOVA followed by Bonferroni's t-test (for three or more groups), comparing all pairs of columns or when appropriate by two-tailed unpaired Student's t-test. Statistical analysis was performed with GraphPad Prism (GraphPad Software, San Diego, CA, USA). For electrophysiology data, the paired sample t-test was used. P values less than 0.05 were considered significant. No statistical methods were used to predetermine sample size. Variation within each data set obtained by experiments with mice or primary cells was assumed to be similar between genotypes since all strains were generated and maintained on the same pure inbred background (C57BL/6).

Study approval

Animal care and handling procedures were in accordance with the International Association for the Study of Pain guidelines for those animals used in pain research and they were approved by the Committee for Ethics in Animal Research of the Ribeirao Preto Medical School -USP (Process nº 045/2013 and 120/2018).

Supplementary references

1. Baban B et al. Indoleamine 2,3-dioxygenase expression is restricted to fetal trophoblast giant cells during murine gestation and is maternal genome specific. *J Reprod Immunol* 2004;61(2):67–77.

2. Parrott JM et al. Neurotoxic kynurenine metabolism is increased in the dorsal hippocampus and drives distinct depressive behaviors during inflammation. *Transl Psychiatry* 2016;6(e918):1–12.

3. Jung S et al. In vivo depletion of CD11c+ dendritic Cells abrogates priming of CD8+ T cells by exogenous cell-associated antigens. *Immunity* 2002;17(2):211–220.

4. Lindquist RL et al. Visualizing dendritic cell networks in vivo. *Nat Immunol* 2004;5(12):1243–1250.

5. Caton ML et al. Notch-RBP-J signaling controls the homeostasis of CD8- dendritic cells in the spleen. *Journal of Experimental Medicine* 2007;204(7):1653–1664.

6. Jung S et al. Analysis of fractalkine receptor CX3CR1 function by targeted deletion and green fluorescent protein reporter gene insertion. *Mol Cell Biol* 2000;20(11):4106–4114.

7. Bourquin A-F et al. Assessment and analysis of mechanical allodynia-like behavior induced by spared nerve injury (SNI) in the mouse. *Pain* 2006;122(14):1–14.

8. Cunha TM et al. A cascade of cytokines mediates mechanical inflammatory hypernociception in mice. *Proc Natl Acad Sci U S A* 2005;102(5):1755–1760.

9. Moriconi A et al. Targeting the minor pocket of C5aR for the rational design of an oral allosteric inhibitor for inflammatory and neuropathic pain relief. *Proc Natl Acad Sci U S A* 2014;111(47):16937–16942.

10. Sant'Anna MB et al. Medial plantar nerve ligation as a novel model of neuropathic pain in mice: Pharmacological and molecular characterization. *Sci Rep* 2016;6(May):1–13.

11. Hargreaves K et al. A new and sensitive method for measuring thermal nociception in cutaneous hyperalgesia. *Pain* 1988;32:77–88.

12. Hunskaar S, Hole K. The formalin test in mice: dissociation between inflammatory and non-inflammatory pain. *Pain* 1987;30(1):103–114.

13. Cunha TM et al. An electronic pressure-meter nociception paw test for mice. *Brazilian Journal of Medical and Biological Research)* 2004;37:401–407.

14. Fonseca MM et al. IL-27 Counteracts Neuropathic Pain Development Through Induction of IL-10. *Front Immunol* 2020;10. doi:10.3389/fimmu.2019.03059

15. Santa-Cecília F V. et al. The NOD2 signaling in peripheral macrophages contributes to neuropathic pain development. *Pain* 2019;160:102–116.

16. Choi Y et al. Behavioral signs of ongoing pain and cold allodynia in a rat model of neuropathic pain. *Pain* 1994;59:369–376.

17. Wilcox GL. Intrathecal morphine in mice: a new technique. *Eur J Pharmacol* 1980;67:313–316.

18. Fujigaki S et al. L-Tryptophan–L-Kynurenine pathway metabolism accelerated by toxoplasma gondii infection is abolished in gamma interferon-gene-deficient mice: cross-regulation between Inducible nitric oxide synthase and indoleamine-2,3-dioxygenase suwako. *Infect Immun* 2002;70(2):779–786.

19. Fuertig R et al. LC – MS / MS-based quantification of kynurenine metabolites , tryptophan , monoamines and neopterin in plasma , cerebrospinal fluid and brain. *Bioanalysis* 2016;8(18):1903–1917.

20. Haque A et al. Induction of apoptosis and immune response by all- trans retinoic acid plus interferon-gamma in human malignant glioblastoma T98G and U87MG cells. *Cancer Immunol Immunother* 2007;56:615–625.

21. Yan Y et al. CNS-specific Therapy for Ongoing EAE by Silencing IL-17 Pathway in Astrocytes. *Molecular Therapy* 2012;20(7):1338–1348.

22. Kohro Y et al. A new minimally-invasive method for microinjection into the mouse spinal dorsal horn. *Sci Rep* 2015;5(14306):1–11.

23. Silva JR et al. Neuroimmune–Glia Interactions in the Sensory Ganglia Account for the Development of Acute Herpetic Neuralgia. *The Journal of Neuroscience* 2017;37(27):6408–6422.

24. Livak KJ, Schmittgen TD. Analysis of relative gene expression data using real-time quantitative PCR and the 2-ΔΔCT method. *Methods* 2001;25(4):402–408.

25. Yogev N et al. Dendritic cells ameliorate autoimmunity in the cns by controlling the homeostasis of PD-1 receptor+ regulatory T cells. *Immunity* 2012;37(2):264–275.

26. Mascanfroni ID et al. Interleukin-27 acts on dendritic cells to suppress the T-cell response and autoimmunity by inducing the expression of ENTPD1 (CD39). *Nat Immunol* 2013;14(10):1054–1063.

27. Ueda H et al. Involvement of lysophosphatidic acid – induced astrocyte activation underlying the maintenance of partial sciatic nerve injury – induced neuropathic pain. *Pain* 2018;159(11):2170–2178.
28. Cardona AE et al. Isolation of murine microglial cells for RNA analysis or flow cytometry. *Nat Protoc* 2006;1(4):1947–1951.
29. Rothhammer V et al. Type I interferons and microbial metabolites of tryptophan modulate astrocyte activity and central nervous system inflammation via the aryl hydrocarbon receptor. *Nat Med* 2016;22(6):586–597.
30. Ting JT et al. Acute brain slice methods for adult and aging animals: application of targeted patch clampanalysis and optogenetics. *Methods in Molecular Biology* 2014;1183:195–204.

Supplementary Figures

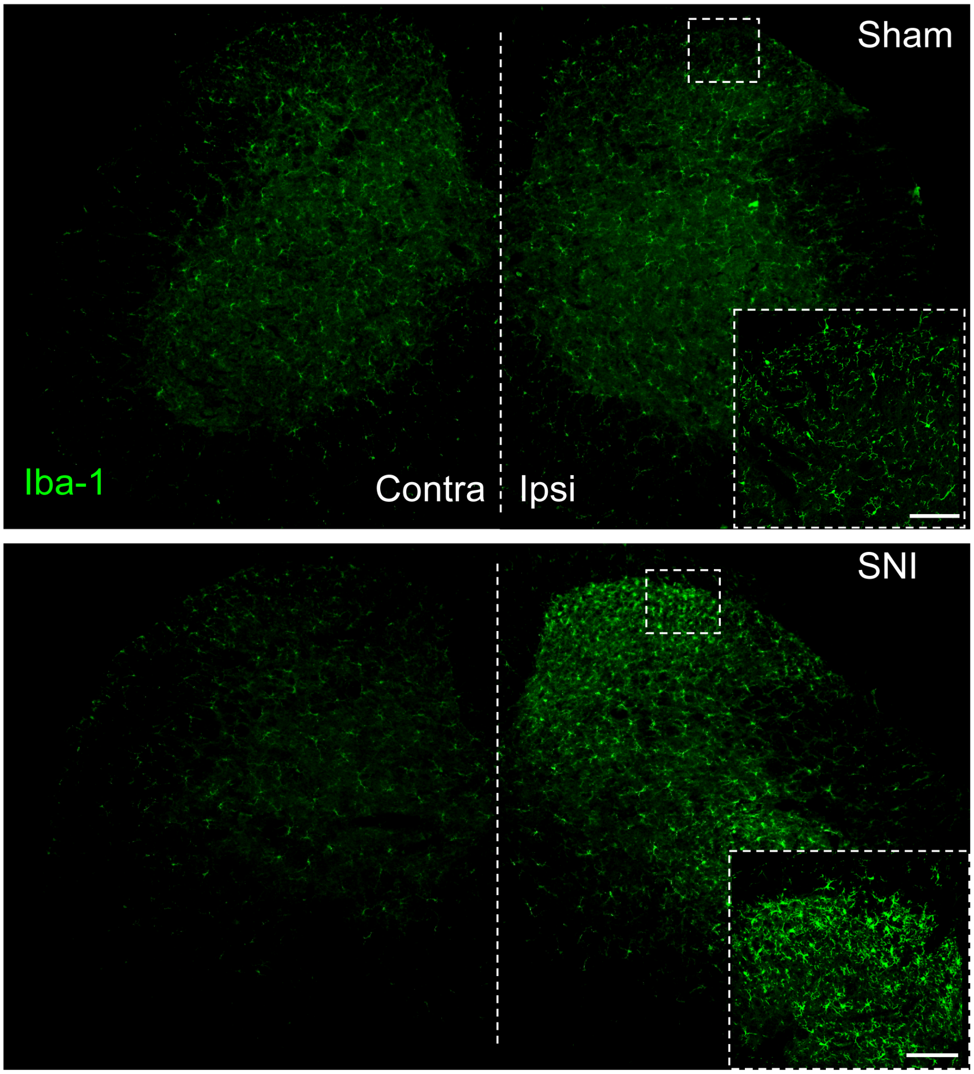

Supplementary Figure 1. Microglial cells activation/proliferation in the spinal cord after spared nerve injury (SNI). Representative confocal images of the spinal cord at 10 days after SNI in WT mice. IBA1+ microglia are shown in green. Dotted boxes show regions of higher magnification in the dorsal horn (right panels). Scale bars: 250 µm (left panels) and 50 µm (right panels).

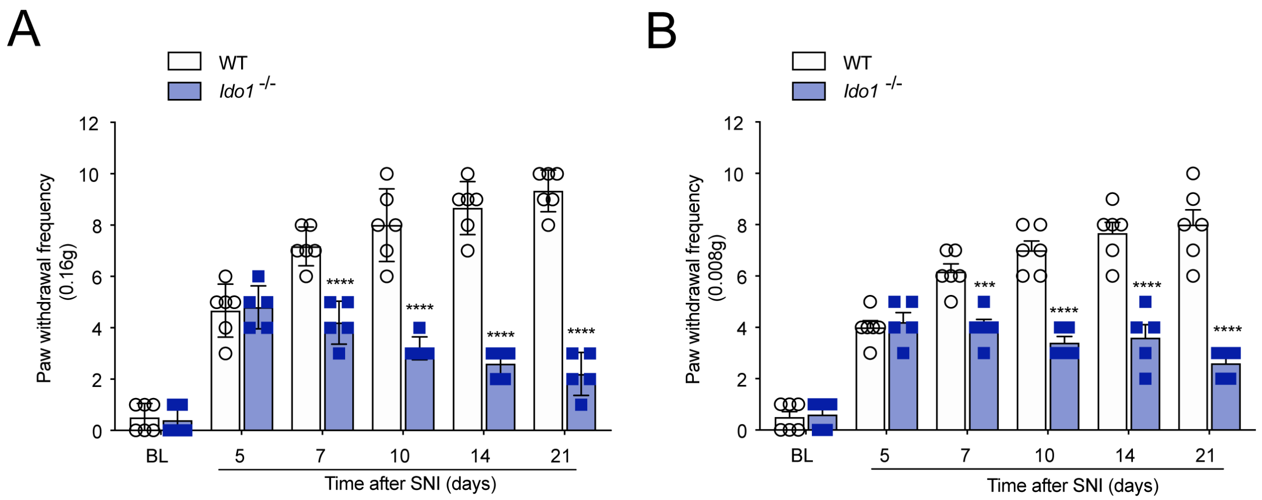

Supplementary Figure 2. The Frequencies of paw withdrawal after hind paw stimulation with von Frey filaments (**A**) 0.16 g or (**B**) 0.008 g. They were evaluated before (BL) and up to 21 days after SNI in WT and *Ido1*$^{-/-}$ mice (n=5-6). Data are expressed as the mean ± s.e.m. ***P < 0.001 and ***P < 0.0001 versus WT group. Two-way ANOVA, Bonferroni's post-test (**A** and **B**).

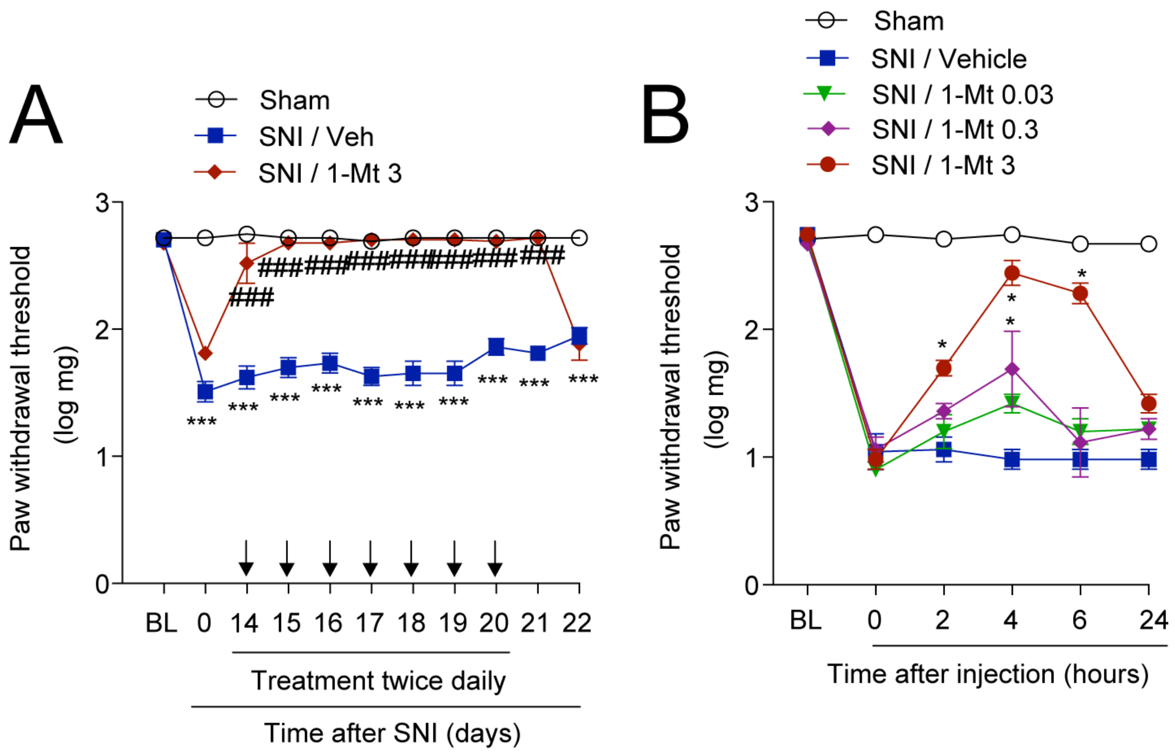

Supplementary Figure 3. (A) Mechanical nociceptive threshold was determined before and 14 days after SNI. Mice were treated (i.p.) with vehicle or 1-Mt 3 mg twice daily during seven consecutive days and mechanical allodynia was measured up to 22 days after SNI (n=6). **(B)** Mechanical nociceptive threshold was determined before and 21 days after SNI. Mice were treated i.p. with vehicle or 1-methil-DL-tryptophan and mechanical allodynia was measured up to 24 h after treatment (1-Mt, 3 mg/per mouse) (n=6). Data are expressed as the mean ± s.e.m. $^*P < 0.05$, $^{***}P < 0.001$ versus sham $^{\#\#\#}P < 0.001$ versus vehicle treated. Two-way ANOVA, Bonferroni's post-test (**A** and B).

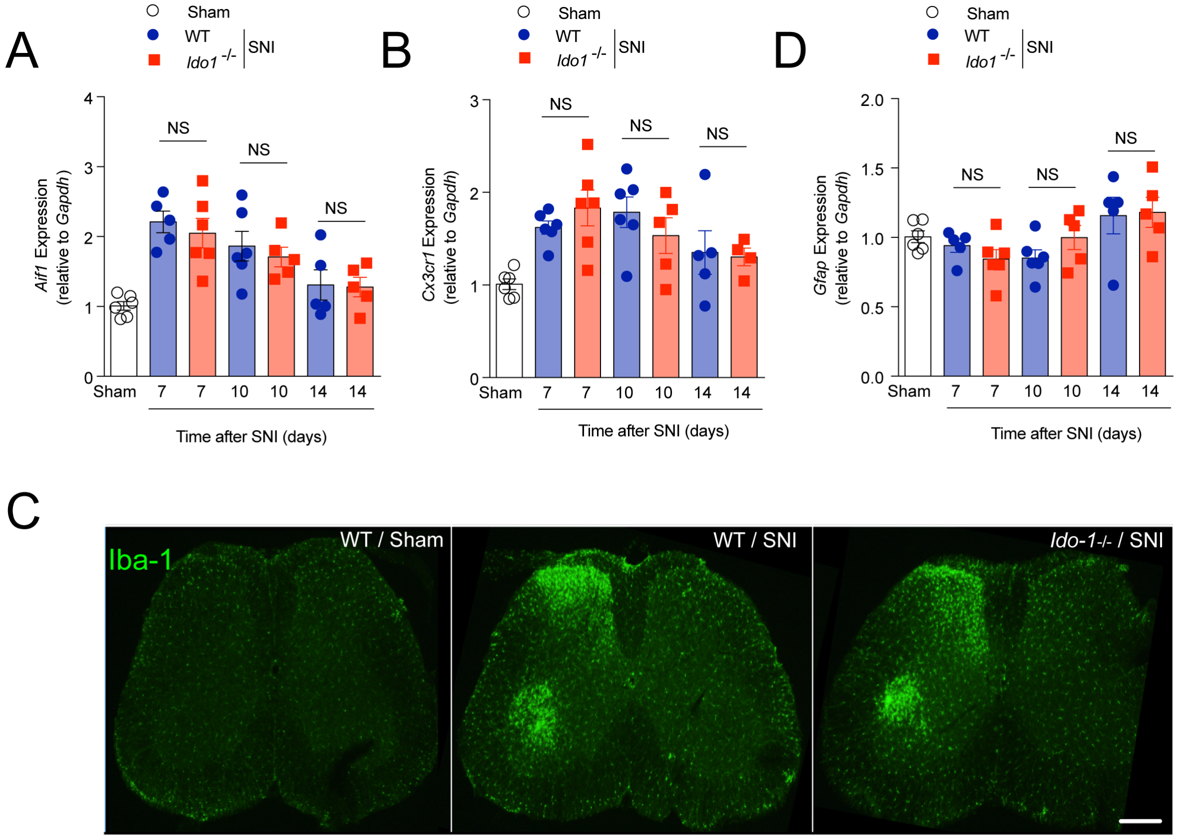

Supplementary Figure 4. Kynurenine metabolic pathway did not participate in the activation of glial cells in the spinal cord after SNI. (A, B and D) The *Aif1, Cx3cr1 and Gfap* mRNA expression in the ipsilateral dorsal horn of the spinal cord (7, 10 and 14 days) after sham or SNI surgeries in WT or *Ido1*$^{-/-}$ mice (n=5-6) **(C)** Representative images showing immunoreactivity for IBA-1 (green color) on spinal cord sections from WT and *Ido1*$^{-/-}$ mice at 10 d after SNI or sham-operated mice (n=3). Scale bars: 300 µm NS = non-significative, One-way ANOVA, Bonferroni's post-test (**A-C**; means ± s.e.m.).

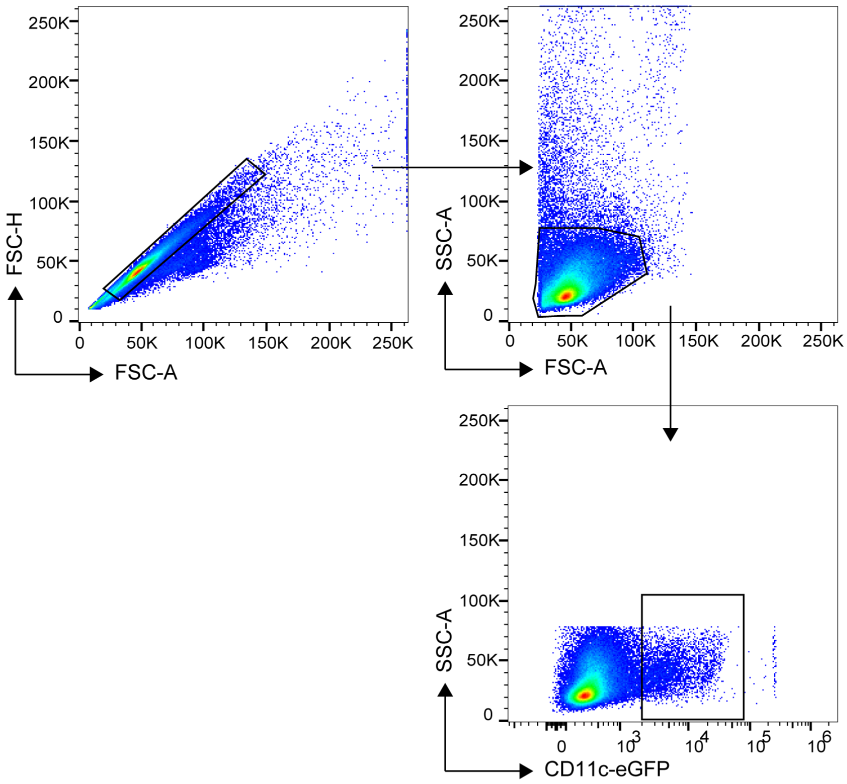

Supplementary Figure 5. Representative FACS sorting strategy for CD11c^{+} cells isolation from draining lymph nodes (popliteal and inguinal).

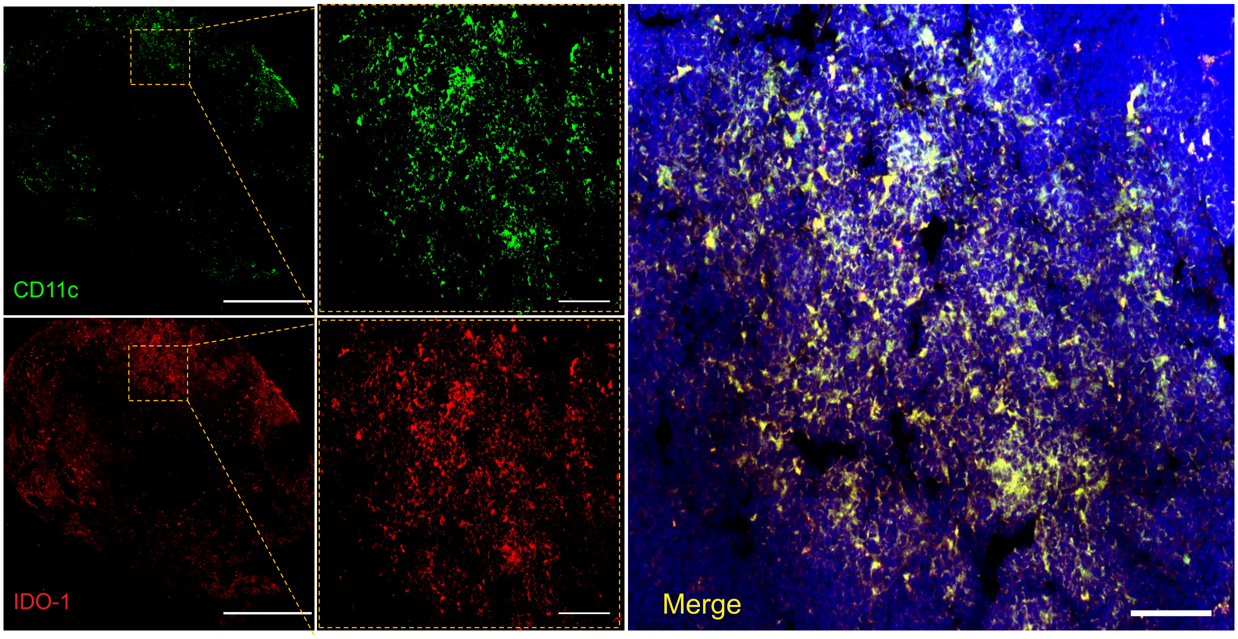

Supplementary Figure 6. **(b)** Representative images showing immunoreactivity for IDO1 (red color) double labeled with anti-CD11c (DCs) in the ipsilateral popliteal lymph node from SNI mice (14 day after SNI). Bar scale 100 µm.

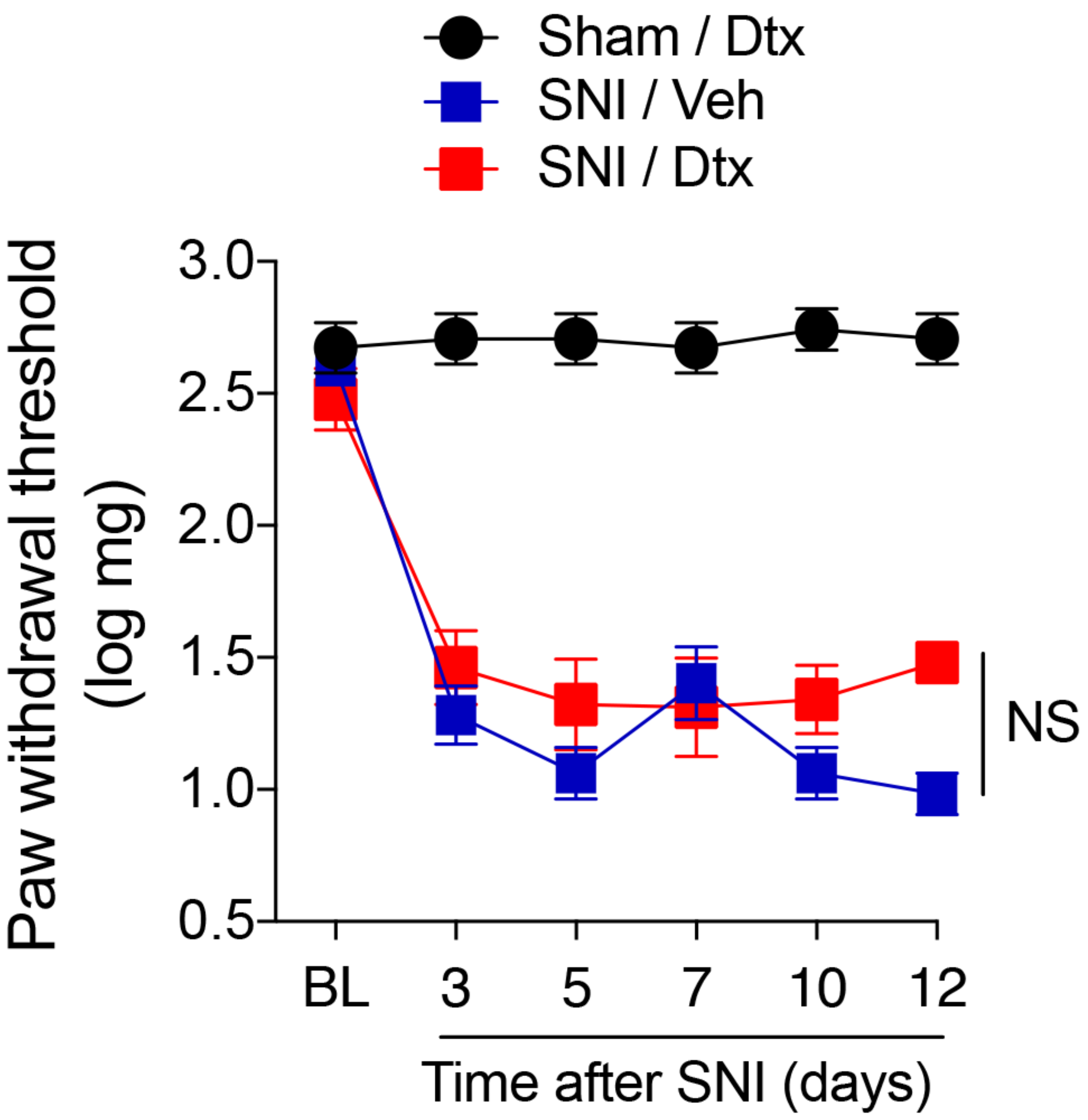

Supplementary Figure 7. **Diphtheria toxin treatment did not change mechanical allodynia in WT mice.** Mechanical nociceptive threshold was determined before and up 21 days after SNI. One group of mice was treated with 16 ng Dtx/g body weight (i.p.) every second day from day 0 up to day 12 after SNI induction (n=6). NS = non-significative; Two-way ANOVA, Bonferroni's post-test (means ± s.e.m.).

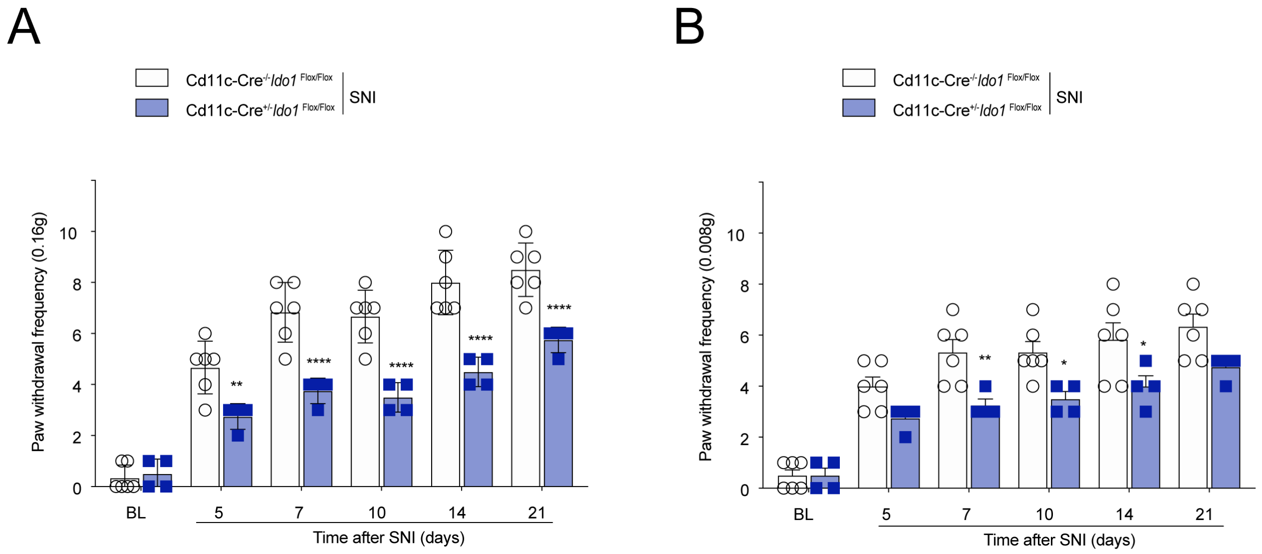

Supplementary Figure 8. The Frequencies of paw withdrawal after hind paw stimulation with von Frey filaments (**A**) 0.16 g or (**B**) 0.008 g. They were evaluated before (BL) and up to 21 days after SNI in WT and *Ido1*$^{-/-}$ mice (n=4-6). Data are expressed as the mean ± s.e.m. **P < 0.05, **P < 0.01 and ****P < 0.0001 versus littermate control. Two-way ANOVA, Bonferroni's post-test (A and B).

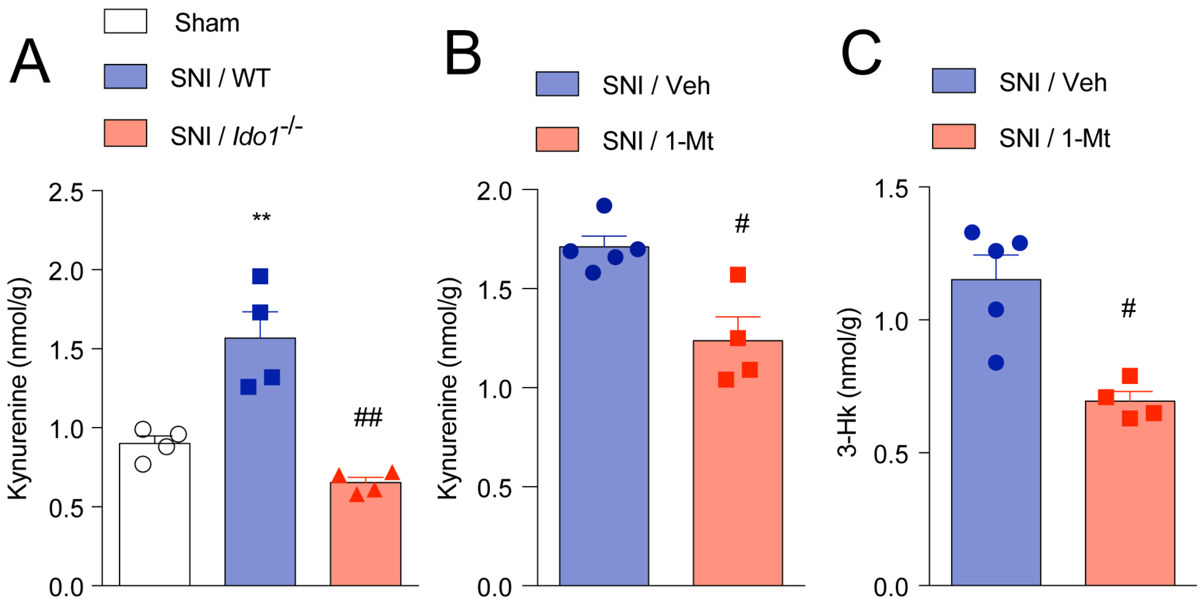

Supplementary Figure 9. Kyn and 3-Hk levels in the spinal cord after SNI: role of IDO-1 expressed in DRLs. Levels of kynurenine (Kyn) was determined in the ipsilateral dorsal horn of the spinal cord of **(A)** WT or *Ido*1$^{-/-}$ mice or in **(B)** WT mice treated with vehicle or 1-methil-DL-tryptophan (1-Mt, 15 µg/per site) after SNI surgeries (14 days after surgeries; n=4-5). **(C)** 3-Hydroxykynurenine (3-Hk) levels in the ipsilateral dorsal horn of the spinal cord of WT mice treated with vehicle or 1-methil-DL-tryptophan (1-Mt, 15 µg/per site) after SNI surgeries (14 days after surgeries; n=4-5) **P*< 0.05 versus sham group. # *P*< 0.05, ## *P*< 0.01 versus vehicle treated. One-way ANOVA, Bonferroni's post-test (**A**; means ± s.e.m.). Unpaired Student's *t*-test (**B** and **C**; means ± s.e.m.).

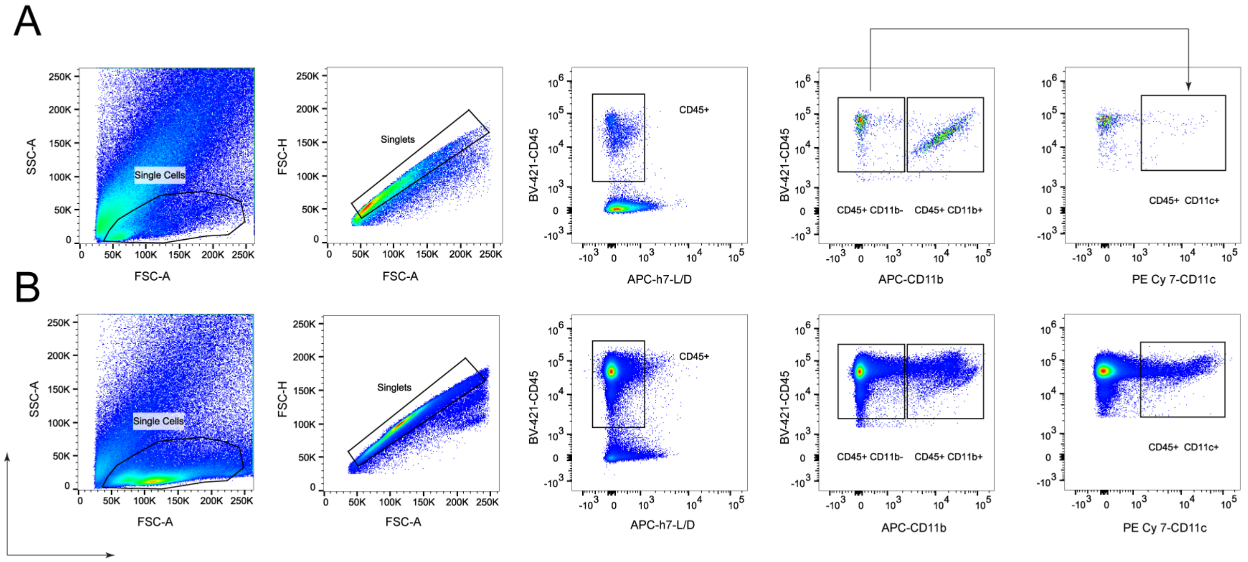

Supplementary Figure 10. Representative gating strategies for flow cytometry analysis from cells in the DRLs. Gating strategies for different cell populations for flow cytometry analyses from (**A**) Sham and (**B**) SNI mice. Side scatter area (SSC-A) and forward scatter area (FSC-A); Doublet cells were excluded by forward scatter height (FSC-H) and FSC-A gating; Viable cells were identified using fixable viability dye and the number of total leukocytes were identified as cells stained for CD45+ among live cells. Myeloid cells were characterized as stained for CD11b+ among CD45+ live cells. Dendritic cells were identified by CD11c+ outside CD11b+ myeloid cells.

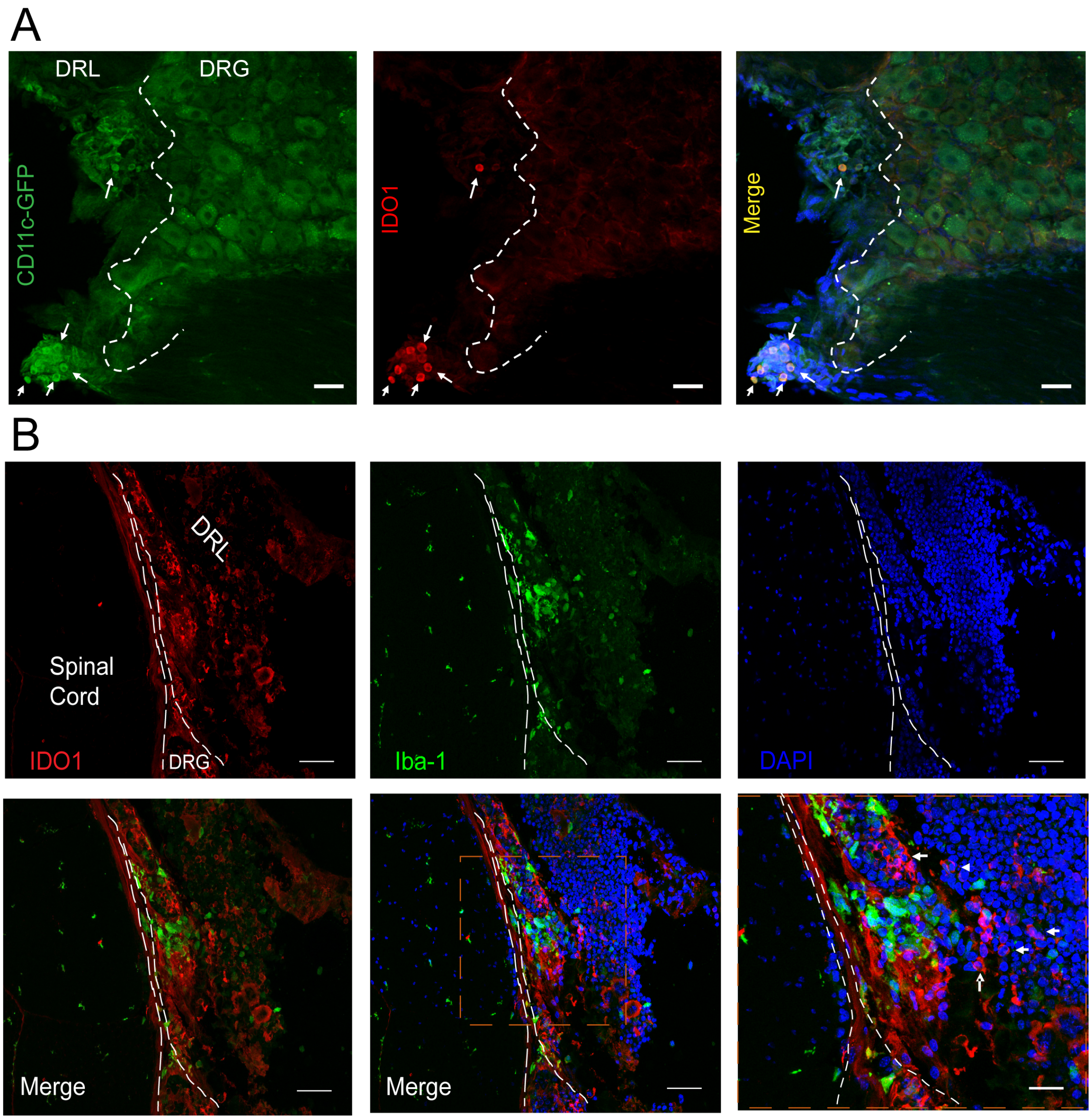

Supplementary Figure 11 (A) Representative images showing immunoreactivity for IDO1 (red color) double labeled with CD11c-eGFP (DCs, white arrow) in the ipsilateral DRGs (L4) from SNI mice (14 day after SNI), bar scale 100 µm. **(B)** Representative images showing immunoreactivity for IDO1 (red color, white arrow) double labeled with IBA-1 (macrophages) in the ipsilateral region containing DRG (L4), DRL and spinal cord from SNI mice (14 day after SNI), bar scale 100 µm.

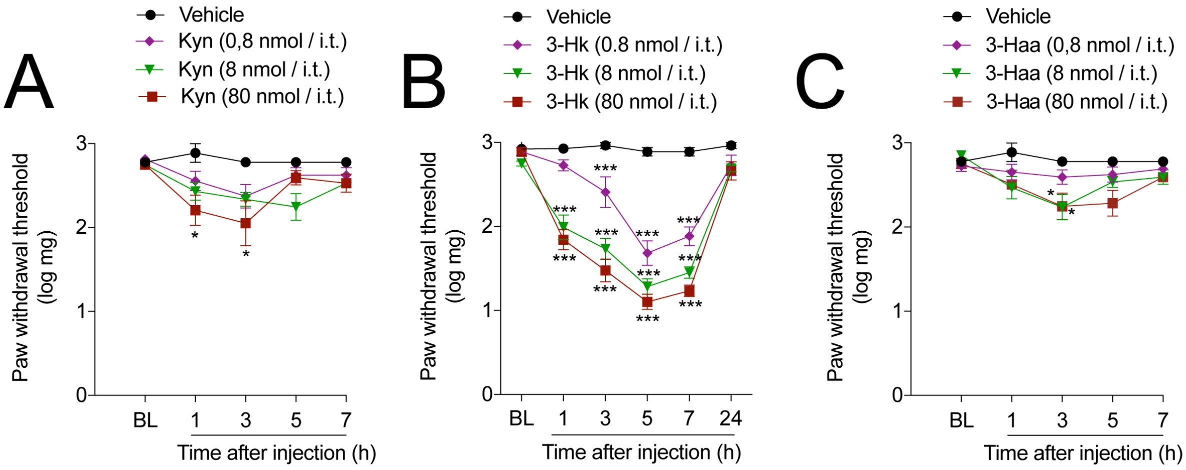

Supplementary Figure 12. Spinal effects of kynurenine metabolites in mechanical nociceptive threshold. Mechanical nociceptive threshold was evaluated before and up to 07 h after intrathecal injection of equimolar doses of (**A**) kynurenine (Kyn, 0.8-80 nmol), (**B**) 3-hydroxykynurenine (3-Hk, 0.8-80 nmol); (**C**) 3-Hydroxyanthranilic acid (3-Haa – 0.8-80 nmol) or vehicle (saline) in WT mice (n=4-6). **P*< 0.05, ****P*< 0.001 versus vehicle treated. Two-way ANOVA, Bonferroni's post-test (means ± s.e.m.).

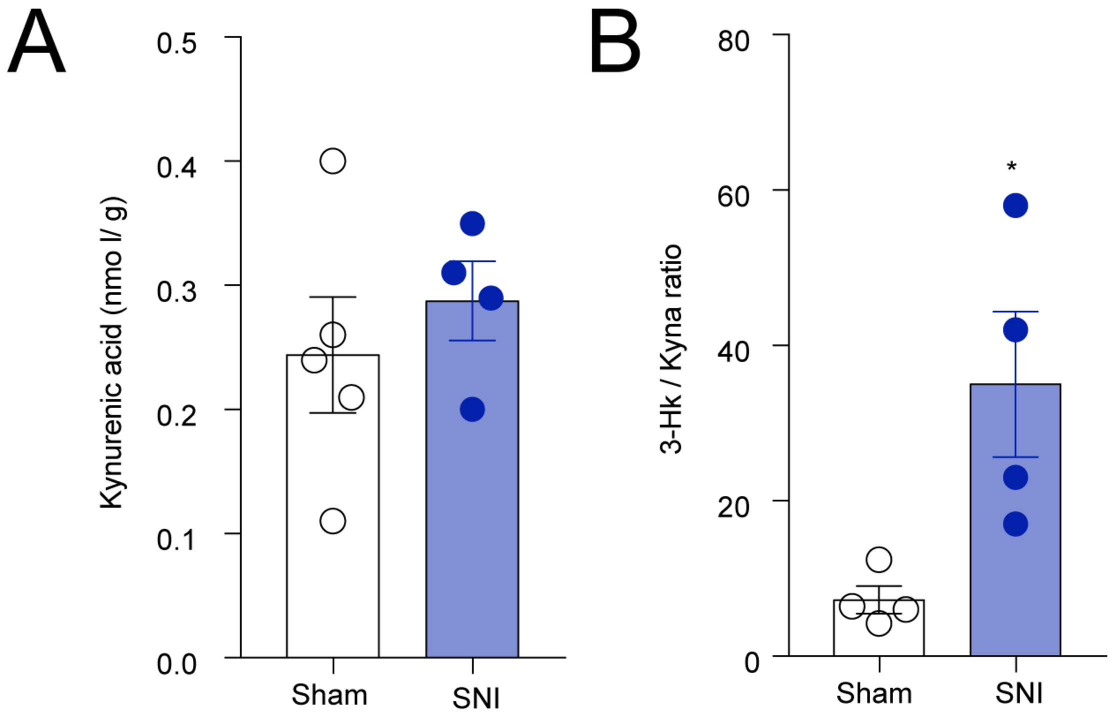

Supplementary Figure 13. Kyna levels and its relationship with 3-Hk in the spinal cord after SNI (A) Levels of kynurenic acid (Kyna) were determined in the ipsilateral dorsal horn of the spinal cord of mice after sham or SNI surgeries (14 days after surgeries; n=4-5). **(B)** Ratio between 3-Hk and Kyna levels in the ipsilateral dorsal horn of the spinal cord of mice after sham or SNI surgeries (14 days after surgeries; n=4). *P< 0.05 versus sham group. Unpaired Student's *t*-test (means ± s.e.m.).

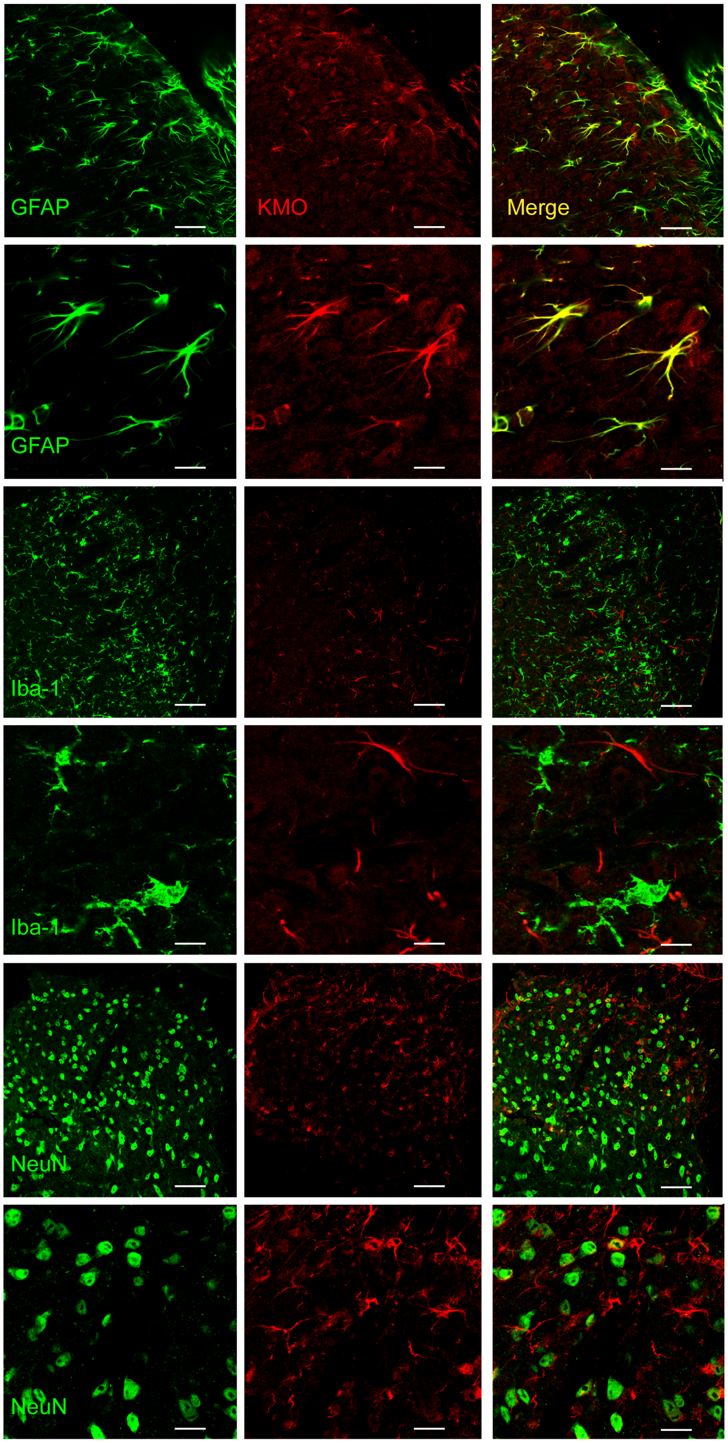

Supplementary Figure 14. Cellular pattern of KMO expression in the spinal cord after peripheral nerve injury. At 14 days after sham or SNI surgeries, the expression of KMO was analyzed by immunofluorescence. Representative of 4, bar scale: 50 and 25 µm.

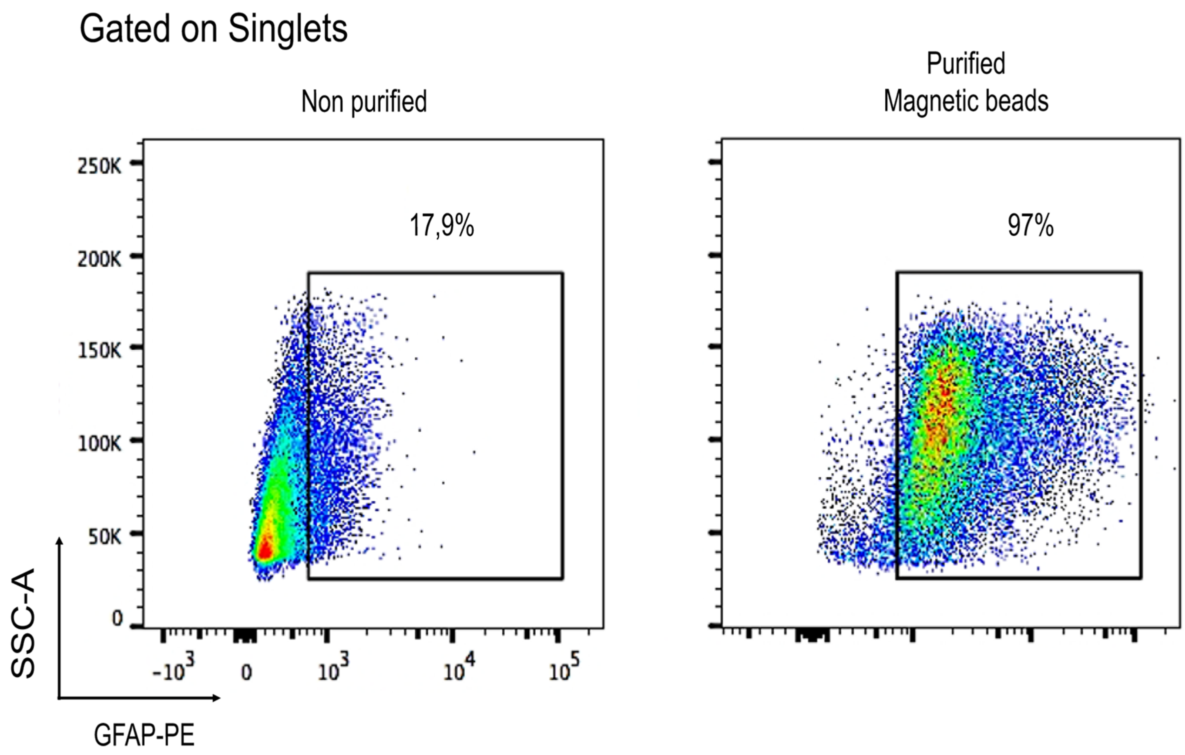

Supplementary Figure 15. Representative FACS strategy for GFAP+ cells showing the efficacy of the purification protocol. Newborn mouse brain cortex cells were isolated and purified as described in the methods section. The FACS panels depicted the percentage of GFAP+ cells before and after purification.

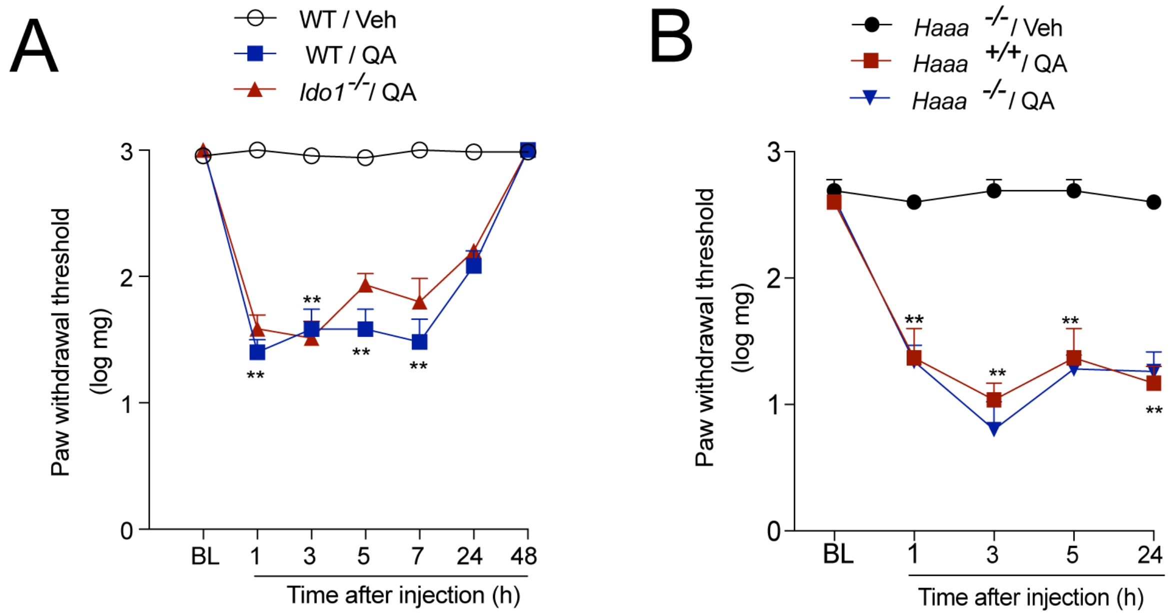

Supplementary Figure 16. Mechanical nociceptive threshold was evaluated before and after intrathecal injection of QA (80 nmol) or vehicle (saline) in WT and **(A)** *Ido1*$^{-/-}$ mice or in **(B)** in *Haao*$^{+/+}$ and *Haao*$^{-/-}$ mice (n=4-5). ***P*< 0.001 versus vehicle treated. NS = non significative. Two-way ANOVA, Bonferroni's post-test (means ± s.e.m.).

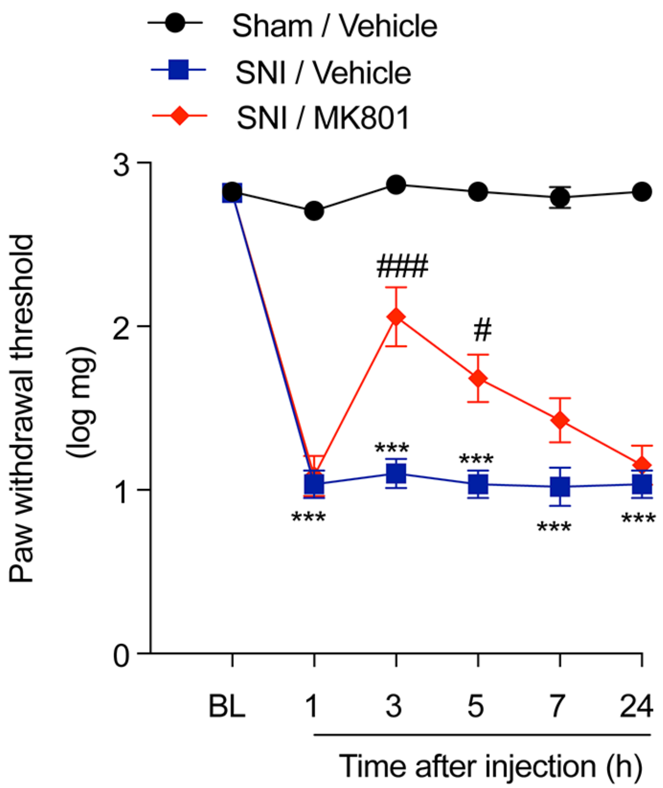

Supplementary Figure 17. NMDA receptors are involved in the maintenance of SNI-induced neuropathic pain. Mechanical nociceptive threshold was determined before and 14 days after SNI. Mice were then treated intrathecally (i.t.) with MK801 (NMDA receptor antagonist, 10 nmol) or vehicle and mechanical allodynia was measured up to 24 h after treatment (n=5-6). ***P< 0.001 versus sham group. $^{\#}P$< 0.05, $^{\#\#}P$< 0.01, SNI vehicle treated. *P< 0.05 versus baseline. Two-way ANOVA, Bonferroni's post-test (means ± s.e.m.).

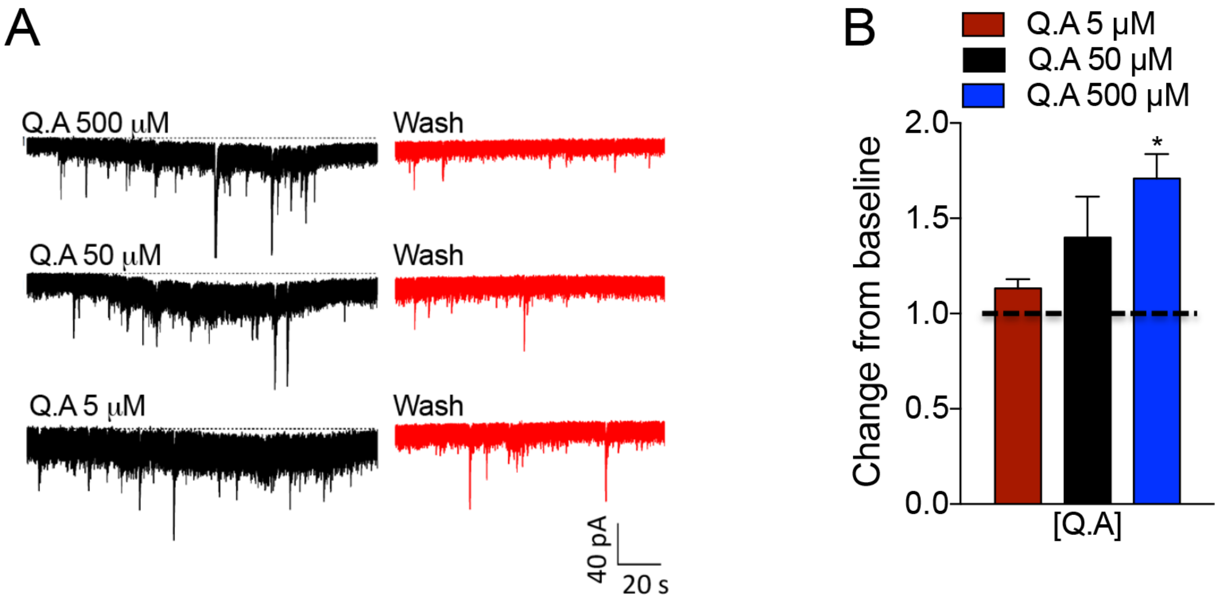

Supplementary Figure 18. QA elicits inward currents in neurons from spinal cord slices (**A**) Representative traces in black show the development of an inward current upon application of QA in different concentrations (5, 50 and 500 µM – black traces) and the drug washout recorded between each application (red traces) (**B**) Current values upon application of QA are shown on the right normalized using the control baseline as reference (n=4). *P< 0.05 versus baseline. The paired t-test (means ± s.e.m.).

Supplementary Table 1. Compound dependent parameters in MRM mode for UPLC®- MS/MS analysis.

Analyte	MRM (*m/z*)	Cone (V)	Collision energy (eV)
Kyn	209 > 94<br>209 > 146<br>209 > 192	16	10
3-Hk	225 > 110<br>225 > 162<br>225 > 208	5	15
Kyna	190 > 89<br>190 > 116<br>190 > 144	26	18
3-Haa	154 > 80<br>154 > 108<br>154 > 136	14	12
TRP_Cl	239 > 180<br>239 > 193<br>239 > 222	10	15

Supplementary Table 2. List of real-time PCR primers

	Gene	Sequence
**Mouse**	*Ido1*	fwd: 5'-TGCCCGACGCATACACC- 3'
		rev: 5'-GTCCGTCCGTGCTCAGTGG- 3'
	Kmo	fwd: 5'-TGTGCCCATGAAAGCCAGAA- 3'
		rev: 5'-GCACCTTCGACTTGGCATAG- 3'
	Haao	fwd: 5'-GCAACAAGCCTTATGCACCAGG- 3'
		rev: 5'-ACATCCACATCCTGCCTTGG- 3'
	Gfap	fwd: 5'-AGGGCGAAGAAAACCGCATCACC- 3'
		rev: 5'-TCTAAGGGAGAGCTGGCAGGGCT- 3'
	Cx3cr1	fwd: 5'-GCCTCTGGTGGAGTCTGCGTG- 3'
		rev: 5'-CGCCCAAATAACAGGCCTCAGCA- 3'
	Aif1	fwd: 5'-TGAGGAGCCATGAGCAGCCAAAG- 3'
		rev: 5'-GCTTCAAGTTTGGACGGCAG- 3'
	Gapdh	fwd: 5'- CATCTTCTTGTGCAGTGCCA- 3'
		rev: 5'- CGGCCAAATCCGTTCAC- 3'

Movies Captions

Movie 1. KMO^+ (red) and $GFAP^+$ (green) cells in the spinal cord

Movie 2. IBA-1^+ microglia (green) and KMO (red)-expressing cells in the spinal cord

Movie 3. $NEUN^+$ neurons (green) and KMO (red)-expressing cells in the spinal cord
